# Presequence-Independent Mitochondrial Import of DNA Ligase Facilitates Establishment of Cell Lines with Reduced mtDNA Copy Number

**DOI:** 10.1371/journal.pone.0152705

**Published:** 2016-03-31

**Authors:** Domenico Spadafora, Natalia Kozhukhar, Mikhail F. Alexeyev

**Affiliations:** 1 Department of Pharmacology, University of South Alabama, 307 University Blvd, Mobile, Alabama, 36688, United States of America; 2 Department of Physiology and Cell Biology, 307 University Blvd, University of South Alabama, Mobile, Alabama, 36688, United States of America; 3 Center for Lung Biology, University of South Alabama, 307 University Blvd, Mobile, Alabama, 36688, United States of America; University of Texas Health Science Center at San Antonio, UNITED STATES

## Abstract

Due to the essential role played by mitochondrial DNA (mtDNA) in cellular physiology and bioenergetics, methods for establishing cell lines with altered mtDNA content are of considerable interest. Here, we report evidence for the existence in mammalian cells of a novel, low- efficiency, presequence-independent pathway for mitochondrial protein import, which facilitates mitochondrial uptake of such proteins as Chlorella virus ligase (ChVlig) and Escherichia coli LigA. Mouse cells engineered to depend on this pathway for mitochondrial import of the LigA protein for mtDNA maintenance had severely (up to >90%) reduced mtDNA content. These observations were used to establish a method for the generation of mouse cell lines with reduced mtDNA copy number by, first, transducing them with a retrovirus encoding LigA, and then inactivating in these transductants endogenous Lig3 with CRISPR-Cas9. Interestingly, mtDNA depletion to an average level of one copy per cell proceeds faster in cells engineered to maintain mtDNA at low copy number. This makes a low-mtDNA copy number phenotype resulting from dependence on mitochondrial import of DNA ligase through presequence-independent pathway potentially useful for rapidly shifting mtDNA heteroplasmy through partial mtDNA depletion.

## Introduction

In most mammalian cells, mitochondria generate the bulk of ATP required to sustain a plethora of diverse cellular processes. Besides generating ATP, mitochondria also play important roles in intracellular calcium signaling [1], apoptosis [2], reactive oxygen species (ROS) production [3], and biosynthesis of haem and iron-sulfur clusters [4, 5]. Mitochondria are unique among organelles of mammalian cells in that they house genetic information in the form of mitochondrial DNA (mtDNA).

Most mitochondrial functions depend, directly or indirectly, on mtDNA, which places it at the center of mitochondrial physiology. Mutations in mtDNA have been implicated in neurodegenerative disorders [6], cancer [7], diabetes [8] and aging [9]. Importantly, alterations in mtDNA copy number can also result in severe disease, such as mtDNA depletion syndromes [10, 11].

Reduction of mtDNA copy number has been reported in mtDNA depletion syndromes [12], in response to mtDNA damage [13], to experimental cerebral ischemia/reperfusion [14], upon intragastric administration of ethanol to experimental animals [15], and in cancer [16, 17], among other conditions. Therefore, availability of tools to engineer cell lines for stable maintenance of altered mtDNA copy number would facilitate studies on understanding cellular effects of changes in mtDNA content, which occur in various pathophysiological conditions. However, no such tools have been reported so far.

Here, we report evidence for the existence, in mitochondria, of a low- efficiency protein import pathway, which facilitates uptake of some proteins lacking conventional matrix targeting sequences (MTS), and demonstrate that this pathway can be used to establish cell lines with reduced mtDNA copy number.

## Materials and Methods

### Cells, viruses and DNA constructs

All cells were propagated in Dulbecco’s Modified Eagle Medium (DMEM) containing 10% Fetal Bovine Serum, 50 μg/ml gentamycin, 50 μg/ml uridine, and 1 mM sodium pyruvate in a humidified atmosphere containing 5% CO_2_ at 37°C, which is permissive for growth of ρ^0^ cells (+UP medium). When indicated, uridine and pyruvate were omitted from this medium for selection of cells containing mtDNA (-UP medium). 3T3#52 is a Tet-On derivative of the NIH 3T3 cell line [18]. 4B6 mouse embryonic fibroblasts were derived from Lig3^flox/flox^ embryos [19]. Plasmids and viral constructs were generated by standard techniques [20] and their diagrams are presented in the S1 Fig.

### Production of virus-containing supernatants and infection of target cells

Retrovirus-containing supernatants were produced by CaPO_4_-mediated transfection of the HEK293FT and Phoenix Ampho cell lines, respectively, using established protocols [21]. Target cells were infected with viruses in 24-well plates or in 35-mm dishes at 20% confluence by incubating them overnight with corresponding supernatant in the presence of 10 μg/mL polybrene (Sigma-Aldrich Corp., St. Louis, MO). The next day, the supernatant was removed and cells were allowed to recover for 24h in DMEM, after which cells were trypsinized, transferred into 150-mm dishes, and antibiotic selection (G418, 1,000 μg/mL; puromycin, 3 μg/mL; hygromycin, 400 μg/mL) was applied for 6 days.

### Western blotting

Protein extracts from treated and control cells were prepared using lysis solution containing 10 mM Tris-HCl, 1% SDS, 1x EDTA-free protease inhibitor cocktail (Roche, Indianapolis, IN). Protein concentrations were measured using the BCA assay (Pierce, Rockford, IL, USA). Proteins were separated by PAAG electrophoresis and transferred to PVDF membranes, blocked and incubated with primary and secondary antibodies using standard techniques [20]. Blots were developed with SuperSignal West Pico and exposed to CL-Xposure film (both Pierce). Primary antibodies were α-HSP60 (mitochondrial, BD Biosciences), α-cytochrome oxidase subunit 1 (AbCam), α-Lig3 and α-MnSOD (BD Biosciences), α-myc Tag (Cell Signaling Technology) and α-TOM-70 (Proteintech).

### Determination of mtDNA copy number

Determination of mtDNA copy number in mouse cells was performed using duplex TaqMan qPCR essentially as described previously [19] using EcoRI-digested total cellular DNA and primers and probes described in the S1 Table. To generate standard curves, a separate linearized calibrator plasmid containing cloned nuclear and mitochondrial targets was used. The 20x qPCR primer mastermix contained 3.33 μM mitochondrial forward and reverse primers, 1.67 μM mitochondrial probe, 13.33 μM nuclear forward and reverse primers, and 6.67 μM nuclear probe.

### CRISPR-Cas9 mediated inactivation of the Lig3

For inactivation of the mouse Lig3 gene by introducing deletions into either exon 1 or exon 8, four plasmids encoding gRNAs were constructed (two for each exon, S1 Table and S1H Fig). After transducing 3T3#52 cells with a retrovirus encoding LigA without MTS, resultant transductants were transfected with four plasmids encoding Cas9, two gRNA plasmids for either exon 1 or exon 8, and EGFP, respectively. EGFP-positive cells were sorted using FACS, plated, and resulting colonies were pre-screened by PCR as described previously [22]. Clones that appeared positive on PCR screen were western blotted for Lig3, and mtDNA copy number was determined in candidate clones by qPCR.

### Determination of the borders of CRISPR-Cas9 induced deletions

Total DNA was extracted from clones, subjected to PCR with primers Ex1F2 plus Ex1R and Ex8F2 plus Ex8R for exons 1and 8, respectively (S1 Table), PCR products were cloned into pBluescriptII SK+ (Stratagene, La Jolla, CA), and transformed into Escherichia coli. Inserts were amplified from twelve E. coli colonies for each 3T3 clone with primers dPvu2 and dPvu3 (S1 Table), and PCR products were sequenced using primers Ex1F2 or Ex8F2 for exons 1 and 8, respectively. Sequence alignment was performed using CLUSTALW, and edited using MS Word.

### Cellular respiration

Oxygen consumption rates in whole attached cells was measured with the help of an XF-24 extracellular flux analyzer (Seahorse Biosciences, Billerica, MA, U.S.A) according to the manufacturer’s recommendations and expressed as pMol/min/μg protein. ATP-linked respiration was determined with the help of oligomycin (OLIG, 5 μM), maximal respiration was induced with Carbonyl cyanide-4-(trifluoromethoxy)phenylhydrazone (FCCP, 1 μM), and non-mitochondrial respiration was determined after injection of rotenone and antimycin A (R+A, 1 μM each).

### Mitochondrial isolation and treatment

Mitochondria were isolated using mitochondria isolation kit for cultured cells (ThermoFisher Scientific) according to manufacturer’s recommendations, except protease inhibitors were omitted. After low-speed centrifugation to remove nuclei, debris, and unbroken cells, mitochondrial suspension was divided into three 400 μl aliquots and either left untreated, or was treated with either 50 or 100 μg/ml Proteinase K for 10 min on ice. After inactivation of Proteinase K with 1mM PMSF (final), mitochondria were pelleted, solubilized in 1x Laemmli buffer, and subjected to SDS/PAAGE.

### Statistical analysis

Pairwise comparisons were performed using the two-tailed unpaired student’s t-test assuming unequal variances. Multiple comparisons were performed using two-way ANOVA followed by Dunnett’s test.

## Results

### In cultured cells, mtDNA copy number varies over 2-fold range

In most population studies mtDNA copy number in a given tissue in apparently healthy individuals varies over 2–10 fold range [23–26], and mtDNA content in the range 40–150% of the average is considered clinically normal [27]. Therefore, we sought to establish mtDNA copy number variability in cultured cells. To this end, we cloned a population of 4B6 mouse embryonic fibroblasts derived from Lig3^flox/flox^ embryos [19], and determined mtDNA copy number in six resulting clones. It varied over 2-fold range, from 56% to 111% of arbitrary chosen clone #1 (S2A Fig). To confirm this observation, we re-cloned clone #1. Again, mtDNA copy number varied over the similar range (S2B Fig).

### Switching mitochondrial DNA ligase alters mtDNA copy number

**Fig 1:**
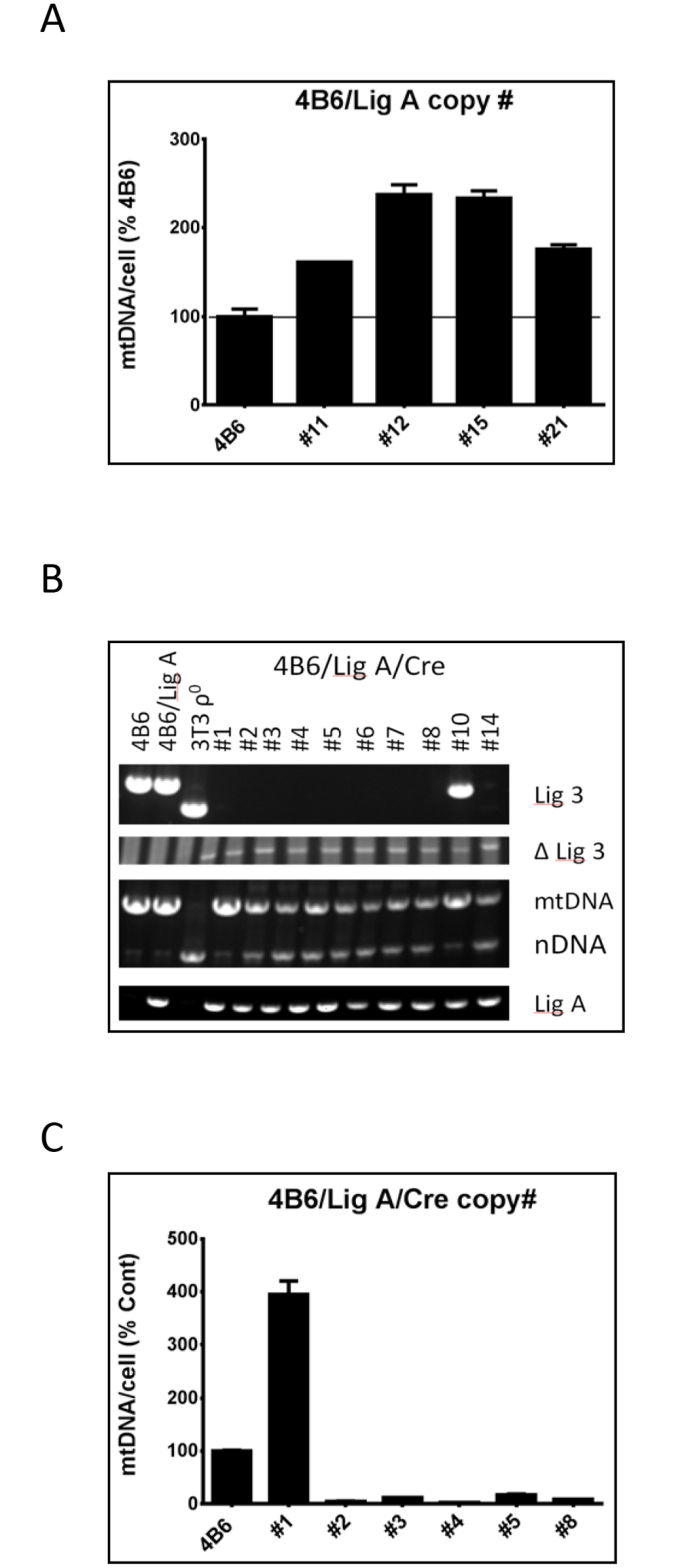
The effect of LigA on mtDNA copy number in 4B6 cells. A, 4B6 cells were transduced with a retrovirus encoding LigA without MTS, and mtDNA copy number was determined in four resulting subclones. B, Subclone #11 was transduced with a retrovirus encoding Cre recombinase, and genomic DNA from ten clones was PCR-analyzed for the presence of unexcised Lig3 allele (Lig3), excised Lig3 allele (ΔLig3), ρ^0^ phenotype (mtDNA and nDNA primers), and for the presence of LigA (LigA). Primer sets and primer sequences are listed in the S1 Table.

DNA ligase III (Lig3) is the only DNA ligase reported in mitochondria. In cultured cells, the loss of Lig3 is accompanied by the loss of mtDNA without the loss of viability [19]. Interestingly, even 100-fold reduced levels of Lig3 in mitochondria are sufficient to maintain mtDNA copy number [19], resulting in the failure of our initial attempts to regulate mtDNA copy number through doxycycline-regulated expression of Lig3 (results not shown). Recently, Simsek et al. reported that both ChVlig and E. coli LigA are able to complement Lig3 deficiency even when expressed without matrix targeting sequences [28]. In silico search for cryptic MTS in these proteins failed to identify potential candidates (results not shown) suggesting that these proteins can be imported through a pathway, which is independent of classical MTS. Because this pathway avoided detection so far, and because most mitochondrial matrix proteins are imported through a canonical MTS-dependent pathway, we hypothesized that this pathway may be less efficient, and that the amount of DNA ligase delivered by this pathway may be insufficient for the maintenance of normal mtDNA copy number. To test this hypothesis, we constructed a retrovirus encoding E. coli LigA gene without an MTS (S1A Fig), and introduced it into 4B6 cells. Unexpectedly, mtDNA copy number in some of the resulting clones was modestly elevated (Fig 1A). Then, clone #11 was transduced with a retrovirus encoding Cre recombinase (S1B Fig). PCR analysis of total DNA from doubly transduced clones revealed that Cre recombinase efficiently induced deletions in the Lig3 gene so that only one of 10 clones tested had incomplete excision (Fig 1B). Subsequent expansion of the nine clones that underwent a complete deletion in all Lig3 alleles and analysis of mtDNA copy number revealed that two clones have lost their mtDNA during expansion and that remaining clones had reduced mtDNA content down to 2% of WT. Surprisingly, one clone, clone #1, had over 3-fold elevated mtDNA content as compared to parental cells (Fig 1C). In subsequent experiments, we were unable to reproduce elevated mtDNA copy number in cells with substituted mitochondrial DNA ligase.

### Reduced mtDNA copy number can be stably inherited in the absence of selection

**Fig 2:**
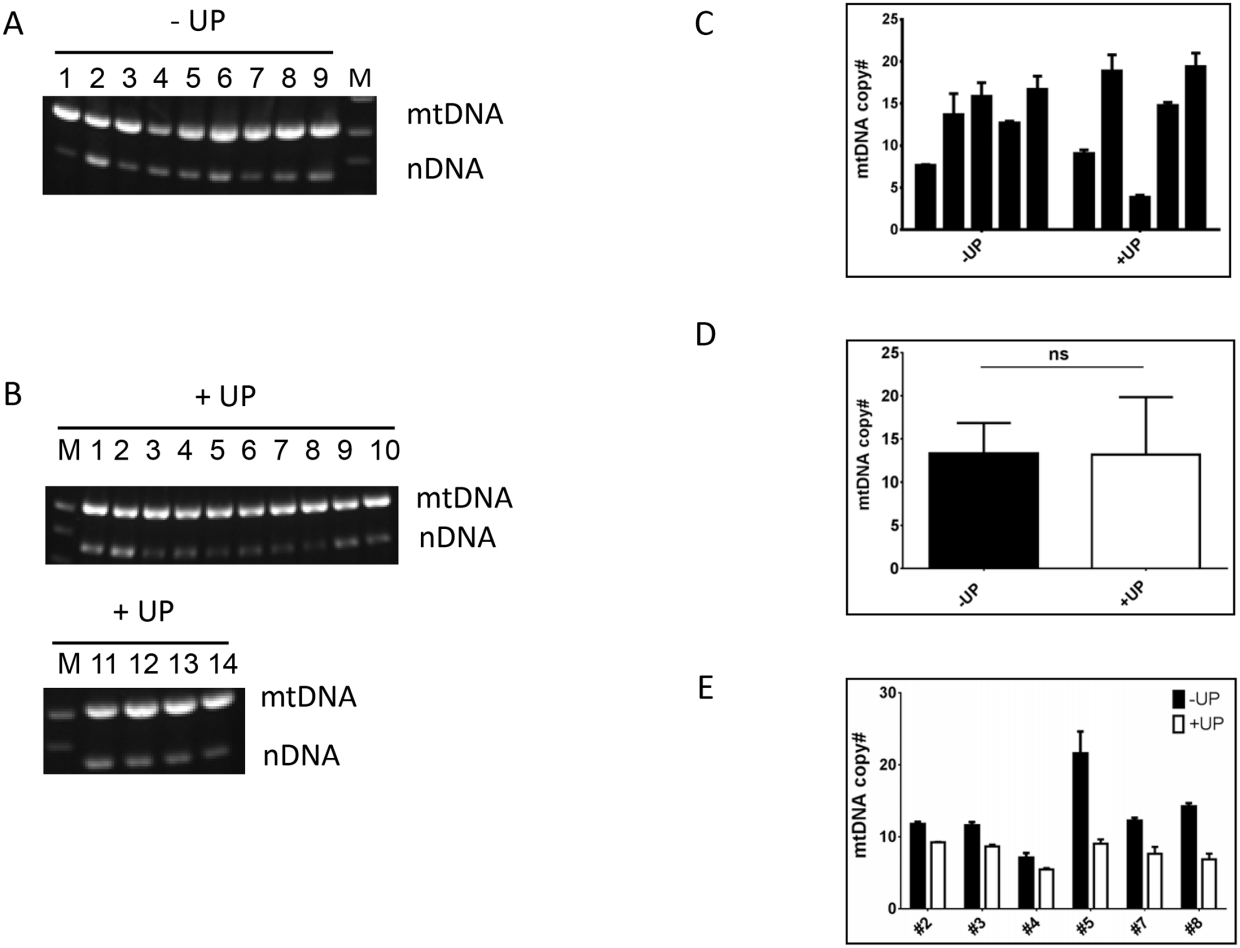
Stability of mtDNA inheritance. Clone #3 was re-cloned in either selective medium devoid of uridine and pyruvate (-UP) or in non-selective medium containing uridine and pyruvate (+UP) after 3 weeks of growth in non-selective medium. Subclones grown in either selective (A) or non-selective (B) medium were tested for the presence of mtDNA by PCR. C. mtDNA copy number was determined in 5 clones grown in either selective (-UP) or non-selective (+UP) conditions, and means were compared (D). ns, not significant, two-tailed Student’s t-test assuming unequal variance. E. mtDNA copy number was determined in six selected clones after 20-day propagation in either selective (-UP) or non-selective (+UP) media.

The loss of mtDNA in two clones during expansion in the medium permissive for the growth of the ρ^0^ cells suggests that in at least some clones with reduced mtDNA copy number LigA-mediated mtDNA maintenance is unstable. This also suggests that low mtDNA copy number observed in most clones may be a consequence of them being mixed populations of cells containing mtDNA and ρ^0^ cells, with predominance of the latter. To experimentally address this possibility, we re-cloned clone #3, in the complete medium, and in the medium devoid of uridine and pyruvate, which is non-permissive for growth of ρ^0^ cells. If mtDNA is unstable in clone #3, then during 3 weeks of this clone propagation in non-selective medium prior to cloning, it would be expected to have accumulated a significant fraction of ρ^0^ cells. In contrast, all clones tested (9 clones grown in selective medium and 14 clones grown in non-selective medium) retained their mtDNA (Fig 2A and 2B). Regardless of the medium used for cloning, mtDNA copy number in clones varied (Fig 2C). However, the average (across all clones tested) mtDNA copy number was not statistically different between clones gown in selective vs. non-selective medium (Fig 2D).

We also conducted a longitudinal study of mtDNA copy number in clones that retained reduced mtDNA copy number during expansion in non-selective conditions. Upon propagation in selective vs. non-selective medium for 20 days, mtDNA content was increased in cells grown in selective medium (Fig 2E). This may indicate preferential growth of cells with increased mtDNA copy number, or low-level instability in the inheritance of mtDNA, which was missed in previous experiments.

### mtDNA copy number depends on activity of DNA ligase and on the ligase import pathway

**Fig 3:**
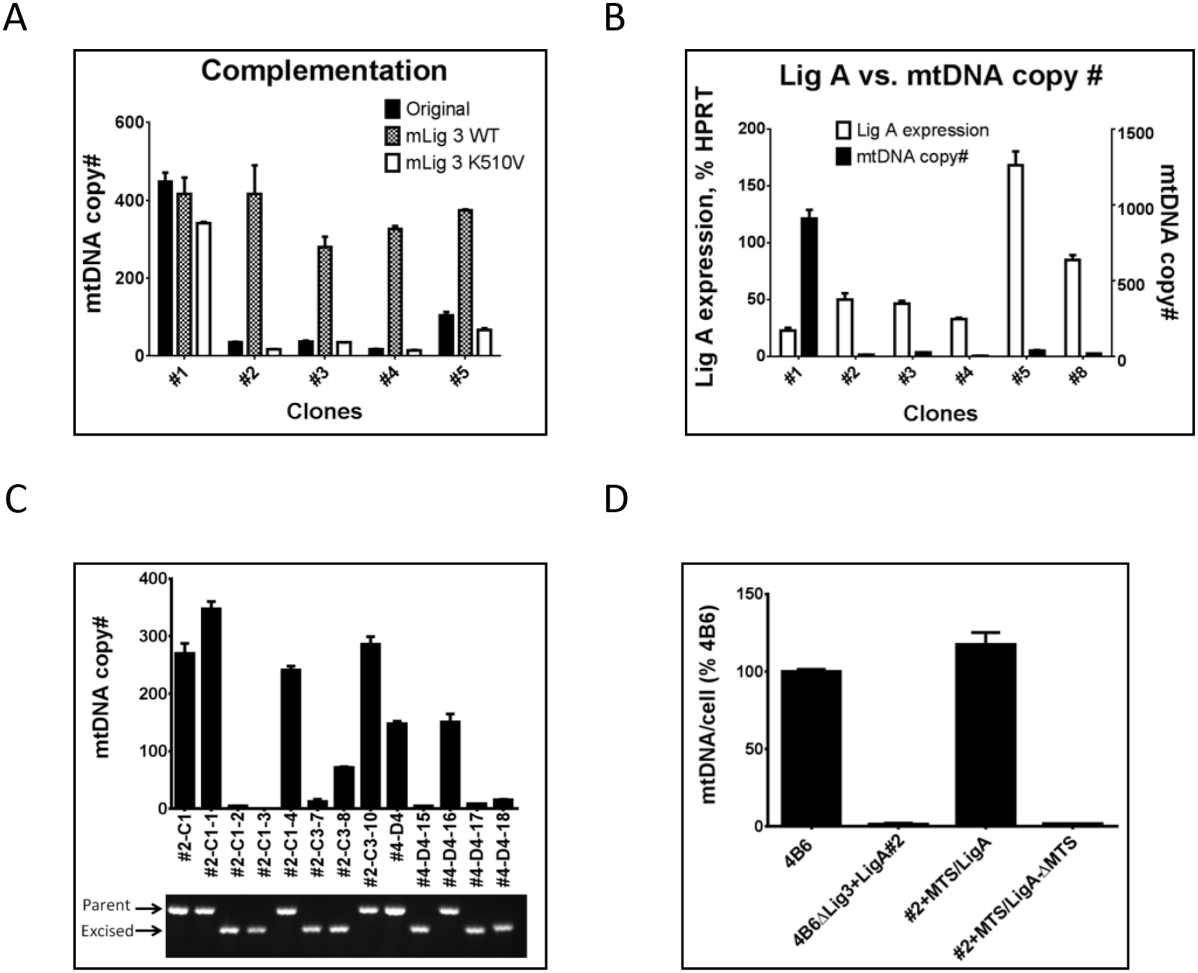
Effects of DNA ligase activity and of the different pathways for mitochondrial import of the LigA on mtDNA copy number. A, transduction with WT, but not catalytically inactive mLig3 restores mtDNA copy number in clones dependent on LigA for mtDNA replication. B, Intracellular levels of the LigA transcript do not directly correlate with mtDNA copy number. C, LigA targeting to mitochondria through the canonical presequence-dependent pathway restores mtDNA copy number in cells dependent on the non-canonical pathway for LigA import. FRT/FLPo recombination-mediated removal of the MTS attached to LigA leads to the reduction in mtDNA copy number. D, Effects of LigA mitochondrial import through different pathways on mtDNA copy number. mtDNA copy number in original 4B6 cells; in clone #2, which lacks Lig3 and supports mtDNA replication by LigA import through the non-canonical pathway; in clone #2, which was complemented with another copy of the LigA targeted to mitochondria through the canonical pathway; and in complemented LigA clone after recombination-mediated removal of the MTS, which ablates LigA uptake through the canonical pathway.

To test whether reduced mtDNA copy number in cells with substitution of the LigA for Lig3 depends on the activity of DNA ligase, we used retroviral transduction to deliver either wild type (WT) or catalytically inactive K510V mutant of the mouse Lig3 (S1 Fig) into WT 4B6 cells and into clones with substitution of the LigA for the Lig3. Transduction with WT mLig3 complemented clones with reduced mtDNA copy number, but had little effect on mtDNA content in clone #1, which has high mtDNA content. In contrast, transduction with catalytically inactive mLig3 had little effect on mtDNA content (Fig 3A). Therefore, reduced mitochondrial content of DNA ligase may be directly responsible for reduced mtDNA copy number.

Collectively, observations that a) LigA is capable of maintaining mtDNA at both high and low copy number and that b) supplementation with WT, but not catalytically inactive, Lig3 restores normal mtDNA levels in clones with low copy number (Fig 3A) raises the question of whether reduced mtDNA copy number is mediated by low LigA expression or by inefficient mitochondrial import of the LigA, since it lacks a canonical MTS. To distinguish between these two possibilities, we used qPCR to determine both mtDNA copy number and levels of the LigA transcript in several clones. Unexpectedly, higher levels of the LigA transcript did not correlate with higher mtDNA copy number (Fig 3B). This observation is consistent with the hypothesis that mtDNA copy number in these clones is limited by mitochondrial import of the LigA.

To independently support the notion that it is inefficient intramitochondrial accumulation of the LigA that is responsible for the reduced mtDNA copy number in clones #2 and #4 (Figs 1C, 3A and 3B), we fused LigA to the MTS from ornithine transcarbamylase (OTC), which was flanked by FRT recombination sites. This fusion can be imported through the canonical, presequence-dependent pathway, and should overcome any limitation that LigA may have in terms of mitochondrial import through a non-canonical pathway. FRT sites facilitate the removal of the MTS OTC through FLPo-mediated recombination and thus enable control for positional effects of retrovirus integration when comparing efficiency of mtDNA maintenance by MTS-containing and MTS-devoid versions of the LigA. The resulting construct containing LigA fused to FRT-flanked MTS OTC was inserted into a retroviral vector (S1E Fig), clones #2 and #4 were transduced with resulting retrovirus, and one subclone from each transduction (2C1 and 4D4, respectively) was selected for transient transfection with a plasmid encoding both optimized FLPo recombinase [29] and red fluorescent protein mCherry [30] (S1G Fig). Cells expressing mCherry were sorted using FACS and plated to form colonies. In colonies formed by cells in which recombination removed MTS OTC (as judged by PCR with primers flanking this structure) mtDNA copy number dropped as compared to parental cells (Fig 3C). In contrast, in colonies, in which recombination failed to remove MTS from LigA, mtDNA copy number remained high (Fig 3C). In summary, when Lig 3 in 4B6 cells was replaced with LigA lacking MTS, mtDNA copy number dropped (clone #2). When this clone was transduced with LigA fused to MTS, mtDNA copy number was restored. Finally, removal of the MTS in a transduced clone with restored mtDNA copy number led to a drop in mtDNA copy number (Fig 3D). These observations strongly support the notion that mtDNA copy number is determined by the mitochondrial import pathway used to deliver LigA to mitochondria.

### Physiological effects of reduced mtDNA copy number

**Fig 4:**
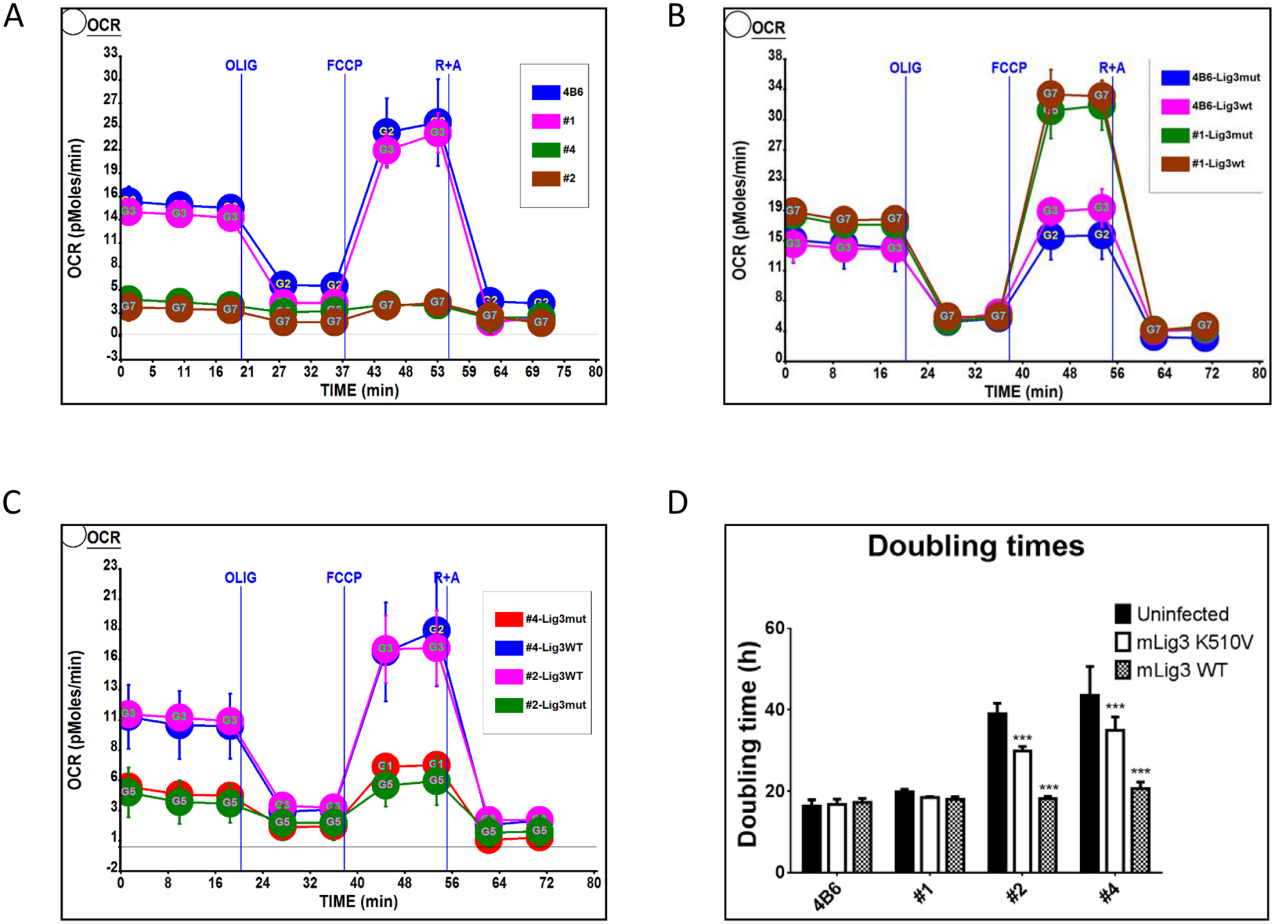
Respiration and growth rates. 4B6 cells and cloned with LigA-supported mtDNA replication #1,#2, and #4 were transduced with retroviruses encoding either WT or catalytically inactive K510V mutant mLig3. A-C, OCR was determined in the parental (A) and transduced (B and C) cells. D, doubling time of the resulting clones. ***, P<0.001, two-way ANOVA with Dunnett’s post-hoc test.

In patients with mtDNA depletion syndromes, mtDNA copy number is severely reduced, and mitochondrial respiratory function is compromised [10]. Yet population studies suggest that mtDNA content in tissues of healthy (and therefore, having normal respiratory function) individuals may vary by as much as 10-fold [23–26, 31]. Moreover, it has been observed that reduced mtDNA copy number has no major effect on mitochondrial transcript levels or enzyme activities in various tissues [32, 33]. Overall, these observations suggest that while, within limits, alterations in mtDNA copy number may have little physiological effect, a severe mtDNA depletion is detrimental. Therefore, we set out to investigate physiological consequences of mtDNA depletion in cell lines, in which mtDNA replication is supported by LigA. First, we measured the baseline mitochondrial respiration in WT 4B6 cells and in clones #1,#2 and #4 (Fig 4A). Despite elevated mtDNA copy number, the oxygen consumption rate (OCR) in clone #1 was the same as in WT 4B6 cells. However, in clones #2 and #4, which have reduced mtDNA copy number baseline respiration was suppressed as compared to WT 4B6 cells (Fig 4A). Introducing either WT or K510V mutant mLig3 into either 4B6 cells, or into clone #1 had little effect on the baseline OCR (Fig 4B). In contrast, transduction of clones #3 and #4 with WT, but not K510V mutant mLig3 led to a significant increase in baseline OCR (Fig 4C), suggesting that reduced mtDNA copy number is responsible for the reduced OCR. Also, empirical observations of variability in growth rates between parental and virus-transduced cell lines suggested precise measurements of proliferation rates. Doubling times in cell lines were inversely proportional to respiration. Transduction of the WT 4B6 cells and a high mtDNA copy number clone #1 with either WT or mutant Lig3 had no effect on growth rates, whereas transduction of low mtDNA copy number clones #2 and #4 with either WT or, surprisingly, with mutant mLig3 resulted in a significant acceleration of growth (Fig 4D).

### ChVlig can support mtDNA replication at reduced copy number

**Fig 5:**
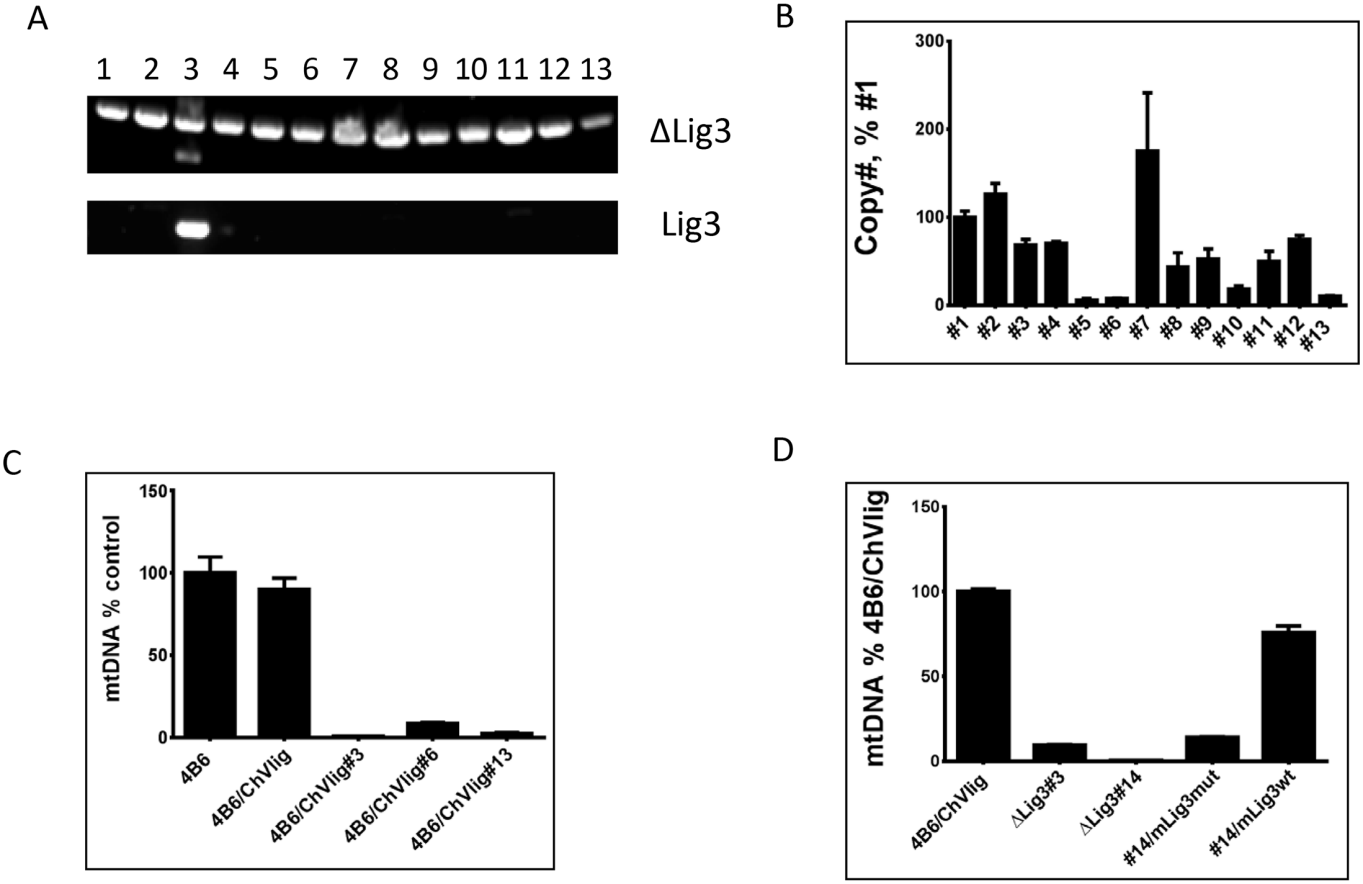
ChVlig can support mtDNA replication at reduced copy number. A, 4B6 cells were sequentially transduced with retroviruses encoding ChVLigAnd Cre recombinase, and Lig3 excision was tested in 13 resulting clones. B, resulting clones have variable mtDNA copy number as compared to an arbitrarily chosen clone#1. C, Clones #5, #6, and #13 maintained >90% reduced mtDNA copy number upon 3-week propagation in the selective medium. D, mtDNA copy number in the 4B6 cells transduced with ChVlig, two clones (#3 and #14), which were derived from the original ChVlig-transduced clone by Lig3 excision, and in the clone#14 after transduction with either WT or catalytically inactive mLig3.

We were further interested in determining whether mitochondrial import of the ChVlig (ChVlig), the smallest known eukaryotic DNA ligase, through an MTS-independent pathway supports mtDNA replication at reduced copy number, as does that of LigA. Unlike the LigA, which uses NAD+ as cofactor [34], ChVlig is similar to the Lig3 in that it uses ATP in DNA end-joining reaction [35]. 4B6 cells were transduced with a retrovirus encoding ChVlig, and one of the resulting clones was re-transduced with a retrovirus encoding Cre recombinase. As in the previous experiment, Lig3 excision was very efficient (12 out of 13 clones tested underwent a complete excision, whereas excision in one clone was incomplete (Fig 5A). The mtDNA copy number in the resulting clones was variable, and reduced by more than 90% in three clones (Fig 5B). These clones retained reduced mtDNA copy number upon a 3-week propagation in the selective medium (Fig 5C). Importantly, WT but not catalytically inactive mLig3 was able restore mtDNA copy number in a clone dependent on the ChVlig for mtDNA replication (Fig 5D). This observation suggests that, similar to LigA, differences in mtDNA content may be mediated by a choice of mitochondrial import pathway.

### Presequence-independent import of the LigA is inefficient

**Fig 6:**
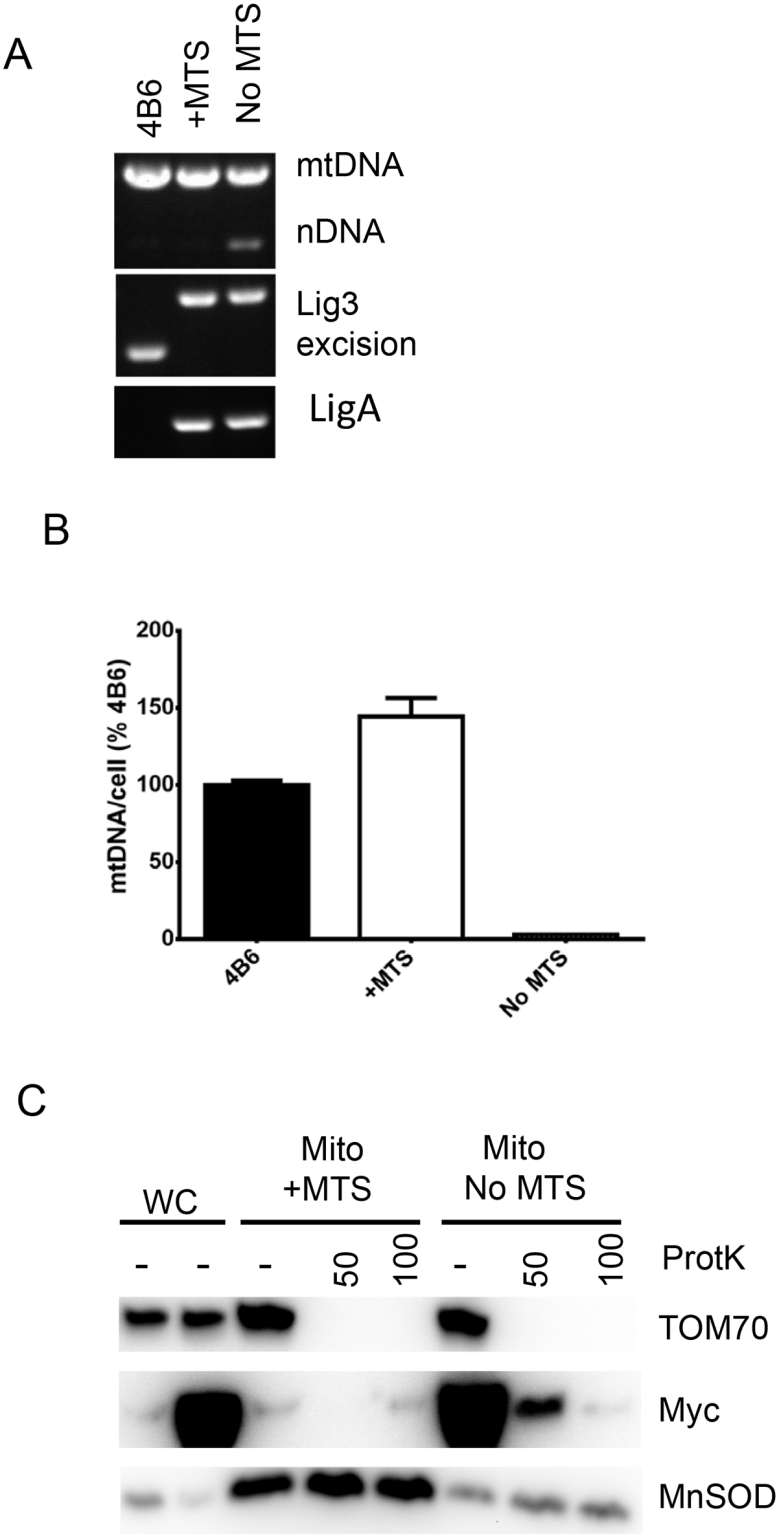
Presequence-independent mitochondrial import of LigA-myc is inefficient. A. Genotyping cell lines transduced with either MTS-LigA-myc or with LigA-myc; B. mtDNA content in the parental 4B6 cells, and in cells in which mtDNA replication is supported by either MTS-LigA-myc (+MTS) or LigA-myc (No MTS); C. Whole cell and mitochondrial fractions from cells that express either MTS-LigA-myc or LigA-myc were subjected to western blotting with myc-tag antibodies, or with antibodies against reference proteins TOM70 (resides in the mitochondrial outer membrane) or MnSOD (resides in the mitochondrial matrix).

To evaluate efficiency of the presequence-independent LigA import, we generated two retroviral constructs encoding C-terminally myc-tagged LigA either fused or not to the OTC MTS (S1 Fig) and established two new 4B6 cell line derivatives, in which mtDNA replication is dependent on these constructs (Fig 6). Remarkably, expression of the mitochondrially targeted LigA-myc as measured in whole cells, was dramatically lower than that of LigA-myc lacking the MTS. Similarly, LigA-myc was barely detectable in mitochondrial preparations from cells expressing MTS-LigA-myc. In contrast, both whole cells lysates and crude mitochondrial preparations from cells expressing LigA-myc without MTS strongly reacted with anti-myc antibodies. However, treatment with Proteinase K destroyed this reactivity suggesting that in the absence of MTS, most of LigA-myc remains outside mitochondria, and that presequence-independent mitochondrial import of LigA-myc is inefficient.

### A method for establishing mouse cell lines with reduced mtDNA copy number

**Fig 7:**
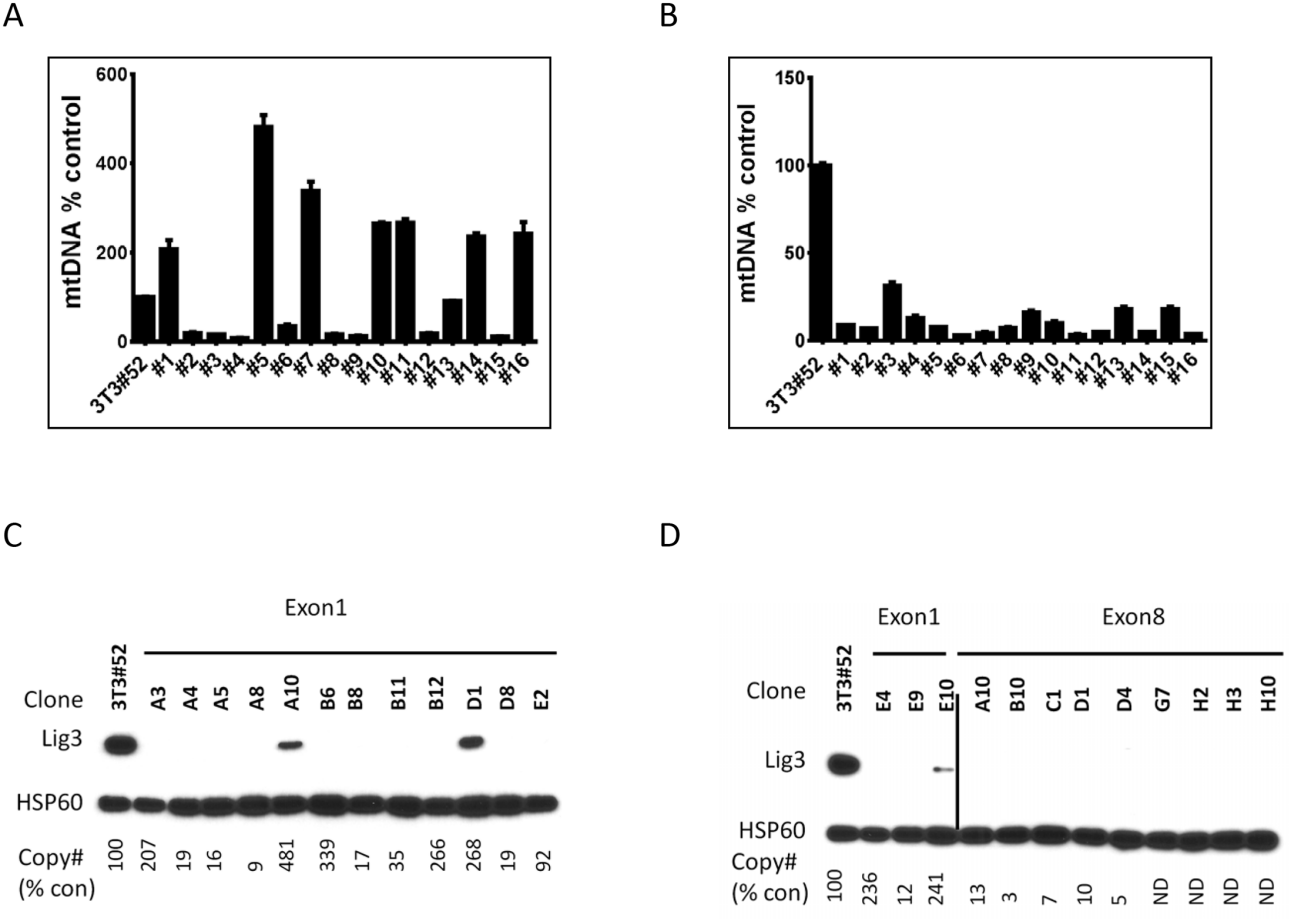
A method for establishing mouse cell lines with reduced mtDNA copy number. A and B, initial screening for mtDNA copy number in 3T3#53 clones expressing LigA, in which Lig3 exon 1 or exon 8 was targeted with CRISPR-Cas9. C and D, The relationship between Lig3 inactivation and mtDNA copy number in clones with targeted exon 1 and exon 8.

The procedure for the establishment of mouse cell lines with reduced mtDNA copy number described above relies on availability of cell lines with floxed Lig3 alleles, and therefore is difficult to generalize. To develop a general method, we designed four single guide RNAs (sgRNAs) for CRISPR-Cas9 mediated inactivation of the Lig3 gene in mouse cells by introducing deletions in exon 1 or in exon 8 of this gene (two sgRNAs for each exon, S1H Fig and S1 Table). The method was validated by inactivating Lig3 in 3T3#52 cells expressing LigA as described in the Materials and Methods. Initial screening of the clones with putative inactivation of the Lig3 revealed substantial heterogeneity in mtDNA copy number in clones in which exon 1 was targeted (Fig 7A). Interestingly, clones which retained Lig3 expression (possibly due to incomplete inactivation of all Lig3 alleles) had high mtDNA content (Fig 7C and 7D), whereas all tested clones with low mtDNA copy number had all Lig3 alleles inactivated (S3B Fig). Of note, clone B6 (Fig 7C), which had high mtDNA content, but no Lig3 detectable by western blotting, had one apparently active allele with two in-frame deletions (S3A Fig). High mtDNA copy number in this clone is consistent with our previous observation that trace amounts of Lig3 are sufficient for the maintenance of normal mtDNA copy number [19]. Remarkably, all tested clones in which exon 8 was targeted had reduced mtDNA copy number (Fig 7B, 7D and S3C Fig). This observation may reflect the fact that exon 8 contains the active site of the enzyme, and therefore both in-frame and out-of-frame deletions in this exon are likely to be detrimental. The reduction in mtDNA copy number in 3T3#52 cells induced by substitution of the Lig3 with LigA was stable (S3D Fig), which underscores the utility of the approach.

### mtDNA depletion with EtBr is accelerated in cell lines with reduced mtDNA copy number

We [18] and others [36] have shown that intracellular mtDNA cloning using partial depletion with EtBr is an effective means of inducing shifts in heteroplasmy (relative abundance of mtDNA haplotypes). However, prolonged treatment with EtBr represents a rate-limiting step of this procedure. Therefore, we were interested whether kinetics of mtDNA depletion in response to EtBr treatment is altered in cells with reduced mtDNA copy number, which is mediated by substitution of the LigA for Lig3. Indeed, in both clones tested mtDNA depletion to the critical threshold of one copy per cell was achieved faster than in parental 3T3#52 cells (7 and 9 days vs. 12 days, respectively). Importantly, mtDNA depletion in cells, which depend on the LigA for mtDNA maintenance was achieved with a lower concentration of EtBr to which parental cells were insensitive (S4 Fig).

## Discussion

Here, we provide initial evidence for the existence of non-canonical low-efficiency pathway for protein import into mitochondria, which facilitates mitochondrial uptake of some proteins lacking mitochondrial presequences, such as E. coli LigA protein and ChVlig. We observed that MTS-LigA targeted for import through canonical presequence-dependent pathway maintains normal mtDNA levels, whereas forcing cells to maintain their mtDNA by means of LigA import through this low-efficiency pathway results in a reduced mtDNA copy number. Together with our previous observation that 100-fold reduced mitochondrial levels of Lig3 are sufficient to maintain normal mtDNA copy number [19], this observation suggests low amount of LigA delivered to mitochondrial matrix through this pathway and therefore inefficiency of this pathway compared to presequence-driven import. Indeed, direct experiments demonstrate that vast majority of the LigA-myc lacking MTS never enters mitochondrial matrix, remains peripherally associated with mitochondria, and is easily digested away with Proteinase K treatment. mtDNA maintenance at low copy number is not an intrinsic property of the NAD+ dependent LigA because ChVlig, which uses ATP rather than NAD+, also maintains mtDNA at low copy number when expressed without MTS. Moreover,

1. mtDNA copy number can be restored in cells, which rely on presequence-independent mitochondrial import of LigA by transducing them with a LigA fused to MTS. In these cells, LigA is imported through both presequence-dependent and -independent pathways;
2. Deletion of the MTS in the resulting cells leads to a drop in mtDNA copy number. This rules out a simple gene dosage effect of LigA fused to MTS.

These experiments unambiguously link low mtDNA copy number to mitochondrial import of the LigA through presequence-independent pathway in cells with inactivated Lig3.

We further describe a means for harnessing this novel pathway by demonstrating that transduction of the target cells with a retrovirus encoding LigA devoid of MTS followed by inactivation of endogenous Lig3 by means of CRISPR-Cas9 RNA-guided nuclease results in the establishment of cell lines with reduced mtDNA copy number. This approach, therefore, can be used as a tool for studying mtDNA copy-number-dependent processes. Cell lines, generated by this approach, also possess reduced growth rate and reduced OCR, both of which can be restored to WT levels by transducing them with WT, but not catalytically inactive encoding DNA ligase fused to an MTS. Interestingly, catalytically inactive mLig3 can partially rescue growth rates and maximal (uncoupled) respiration in these cells suggesting possible mtDNA maintenance-independent roles for Lig3 in cell cycle regulation and ETC function.

Interestingly, mtDNA depletion to an average level of one copy per cell proceeds faster in cells engineered to maintain mtDNA at low copy number. This makes a low-mtDNA copy number phenotype resulting from dependence on mitochondrial import of DNA ligase through presequence-independent pathway potentially useful for rapidly shifting mtDNA heteroplasmy through partial mtDNA depletion.

In this study, we observed increased mtDNA copy number in response to different manipulations. Unexpectedly, one of the 4B6 clones in which mtDNA replication is supported by LigA without MTS had increased mtDNA copy number. Even though we were unable to reproduce this phenomenon in subsequent experiments, we believe that this phenomenon deserves further investigation. Some 4B6 and 3T3 clones expressing both Lig3 and LigA had elevated mtDNA copy number. The magnitude of this increase, however, was much smaller than the magnitude of the drop in mtDNA copy number observed in clones with decreased mtDNA copy number. Considering the modest magnitude of this increase and spontaneous fluctuations of mtDNA copy number in cultured cells over at least twofold range, we have to defer detailed studies to properly document manipulations that increase mtDNA copy number and elucidation of possible mechanism(s) of such increase(s), to future studies.

## Supporting Information

S1 Fig Vector maps. A, retrovirus encoding Escherichia coli LigA. B, retrovirus encoding Cre recombinase; C and D, retroviruses encoding WT and K510V mutant mouse Lig3; E, a retroviris encoding LigA fused to MTS OTC flanked by FRT sites; F, a retrovirus encoding the ChVlig; G, a plasmid encoding FLPo and mCherry proteins; H, a plasmid for expression of sgRNAs to either exon 1 or exon 3 of the mouse Lig3; I, a retrovirus encoding a myc- = tagged LigA with MTS; J, a retrovirus encoding amyc-tagged LigA without MTS. Abbreviations: amp, bacterial ampicillin resistance gene; BGH pA, SV40 pA, corresponding viral polyadenylation signals; ChVlig, ChVlig; PGK, RSV, SV40 and U6, corresponding promoters; Cre, bacteriophage P1 Cre recombinase; F1 ori, single-stranded origin of replication of the bacteriophage F1; FRT, recognition sites for Flp recombinase; FLPo, optimized FLP recombinase gene; GAG, retroviral GAG protein; Hph, hygromycin phosphotransferase, hygromycin resistance gene; IRES, internal ribosome entry site; mLig3 WT, wild type mouse DNA ligase III; mLig3K510V; catalytically inactive mouse DNA ligase III; LigA, Escherichia coli DNA ligase A gene; LTR, long terminal repeat; mCherry, Red fluorescent protein mCherry; MTS, mitochondrial matrix targeting sequence of human ornithine transcarbamylase (1); Myc, myc-tag; Neo, G418 and kanamycin resistance gene; ori, bacterial origin of replication; WPRE, woodchuck hepatitis virus posttranscriptional regulatory element.
(PPTX)

S2 Fig Variability of mtDNA copy number in cultured cells. A, 4B6 cells were cloned, and mtDNA copy number was determined in six resulting subclones. B, subclones #1 was re-cloned, and mtDNA copy number was determined in 5 resulting subclones.
(PPTX)

S3 Fig Deletions in the Lig3 gene induced by CRISPR/CAS9. A, Deletions in the Lig3 exon 1 found in a clone with elevated mtDNA copy number. B and C, Deletions in the Lig3 exons 1 and 8, respectively, found in clones with reduced mtDNA copy number. Blue and underlined are gRNA targets, purple and underlined, sequences from an allele containing two in-frame deletions. Ter, premature translation termination, green and underlined AAG, a codon for active site lysine in exon 8. H, Reduced mtDNA copy number phenotype is stable over at least 3 weeks in clones with targeted exon 8. Clones #1, 2, 3 and 4 (Fig 6B) were grown in media supplemented with uridine and pyruvate, and mtDNA copy number was re-measured.
(PPTX)

S4 Fig mtDNA depletion is accelerated in clones with reduced mtDNA content. Parental 3T3#52 and its derivatives D4 and E9, in which mtDNA replication is supported by LigA were grown in the presence of indicated EtBr concentrations. A fraction of cells was removed at regular intervals, and mtDNA copy number was determined by qPCR.
(PPTX)

S1 Table Oligonucleotides. (DOC)
