# Correction: Presequence-Independent Mitochondrial Import of DNA Ligase Facilitates Establishment of Cell Lines with Reduced mtDNA Copy Number

**DOI:** 10.1371/journal.pone.0156168

**Published:** 2016-05-24

**Authors:** Domenico Spadafora, Natalia Kozhukhar, Mikhail F. Alexeyev

Panel B is erroneously a duplicate of Panel A in S2 Fig. Please view the correct S2 Fig here.

## Supporting Information

S2 Fig Variability of mtDNA copy number in cultured cells. A, 4B6 cells were cloned, and mtDNA copy number was determined in six resulting subclones. B, subclones #1 was re-cloned, and mtDNA copy number was determined in 5 resulting subclones.
(PPTX)
