# Supplementary material for: Presequence-Independent Mitochondrial Import of DNA Ligase Facilitates Establishment of Cell Lines with Reduced mtDNA Copy Number
Source: PLoS One. 2016 Mar 31;11(3):e0152705. doi: 10.1371/journal.pone.0152705 (PMC4816344; doi:10.1371/journal.pone.0152705)
Supplement: S1 Fig — A, retrovirus encoding Escherichia coli LigA. B, retrovirus encoding Cre recombinase; C and D, retroviruses encoding WT and K510V mutant mouse Lig3; E, a retroviris encoding LigA fused to MTS OTC flanked by FRT sites; F, a retrovirus encoding the ChVlig; G, a plasmid encoding FLPo and mCherry proteins; H, a plasmid for expression of sgRNAs to either exon 1 or exon 3 of the mouse Lig3; I, a retrovirus encoding a myc- = tagged LigA with MTS; J, a retrovirus encoding amyc-tagged LigA without MTS. Abbreviations: amp, bacterial ampicillin resistance gene; BGH pA, SV40 pA, corresponding viral polyadenylation signals; ChVlig, ChVlig; PGK, RSV, SV40 and U6, corresponding promoters; Cre, bacteriophage P1 Cre recombinase; F1 ori, single-stranded origin of replication of the bacteriophage F1; FRT, recognition sites for Flp recombinase; FLPo, optimized FLP recombinase gene; GAG, retroviral GAG protein; Hph, hygromycin phosphotransferase, hygromycin resistance gene; IRES, internal ribosome entry site; mLig3 WT, wild type mouse DNA ligase III; mLig3K510V; catalytically inactive mouse DNA ligase III; LigA, Escherichia coli DNA ligase A gene; LTR, long terminal repeat; mCherry, Red fluorescent protein mCherry; MTS, mitochondrial matrix targeting sequence of human ornithine transcarbamylase (1); Myc, myc-tag; Neo, G418 and kanamycin resistance gene; ori, bacterial origin of replication; WPRE, woodchuck hepatitis virus posttranscriptional regulatory element. (PPTX) [file pone.0152705.s001.pptx]

## Slide 1
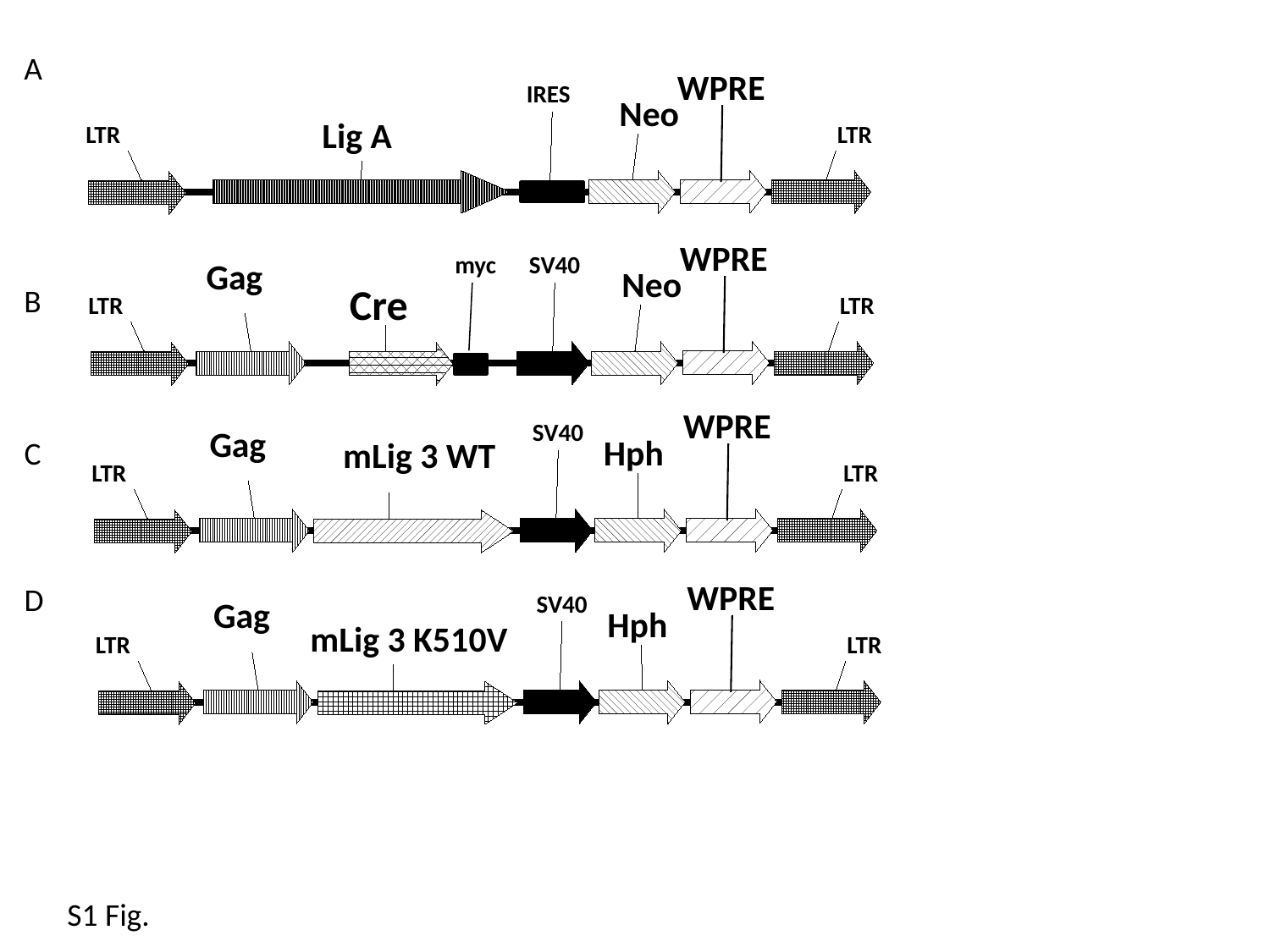

A
WPRE
IRES
Neo
Lig A
LTR
LTR
WPRE
myc
SV40
Gag
Neo
Cre
LTR
LTR
B
WPRE
SV40
Gag
Hph
mLig 3 WT
LTR
LTR
C
WPRE
SV40
Gag
Hph
mLig 3 K510V
LTR
LTR
D
S1 Fig.

## Slide 2
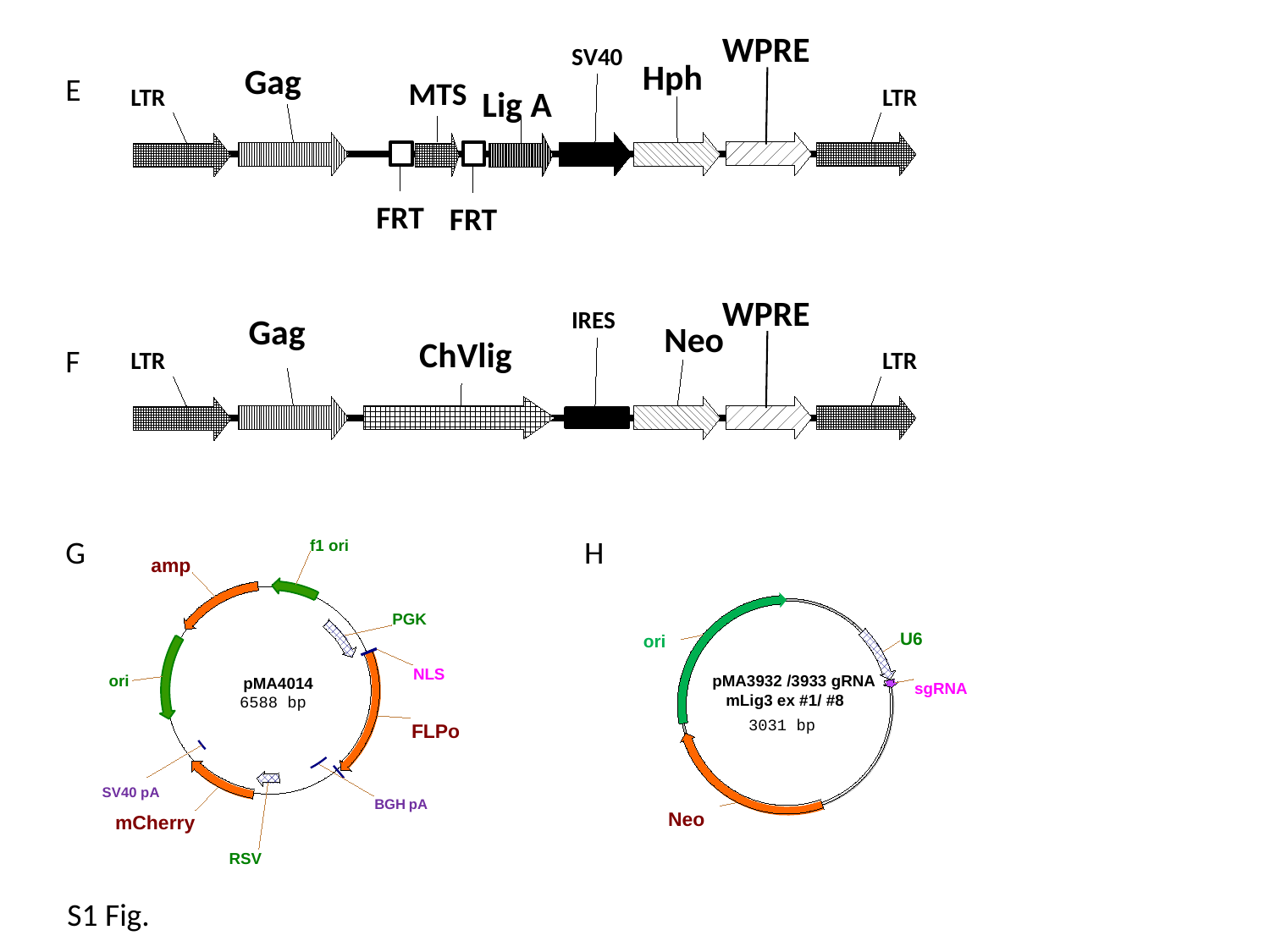

WPRE
SV40
Hph
Gag
MTS
LTR
LTR
Lig A
FRT
FRT
E
WPRE
IRES
Gag
Neo
ChVlig
LTR
LTR
F
G
H
f1 ori
amp
PGK
NLS
ori
pMA4014
6588 bp
FLPo
SV40 pA
BGH pA
mCherry
RSV
U6
ori
pMA3932 /3933 gRNA
 mLig3 ex #1/ #8
sgRNA
3031 bp
Neo
S1 Fig.

## Slide 3
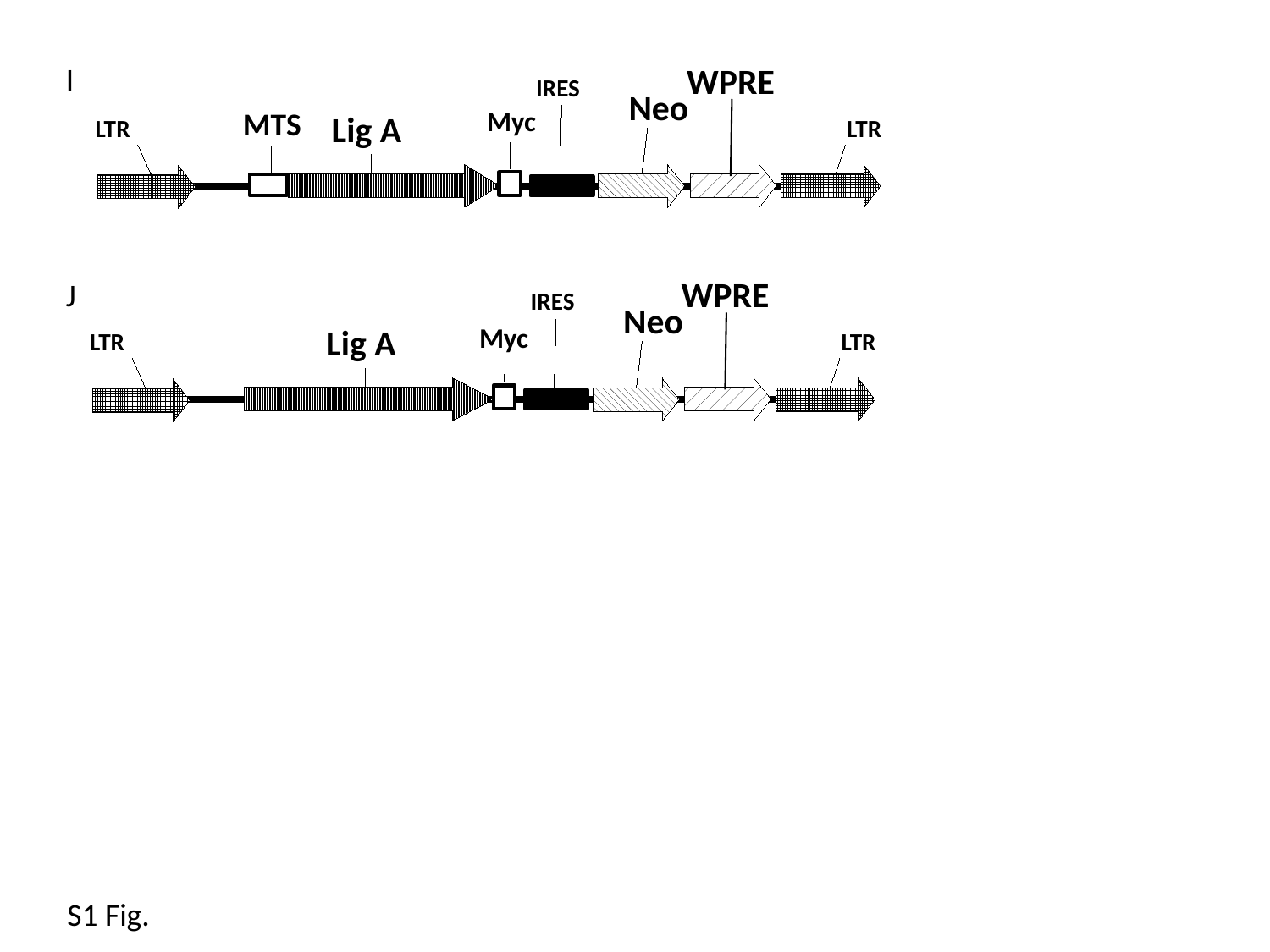

I
WPRE
IRES
Neo
Lig A
LTR
LTR
Myc
MTS
WPRE
J
IRES
Neo
Myc
Lig A
LTR
LTR
S1 Fig.
