# Supplementary figures and images for: Presequence-Independent Mitochondrial Import of DNA Ligase Facilitates Establishment of Cell Lines with Reduced mtDNA Copy Number
Source: PLoS One. 2016 Mar 31;11(3):e0152705. doi: 10.1371/journal.pone.0152705 (PMC4816344; doi:10.1371/journal.pone.0152705)

## Slide 1
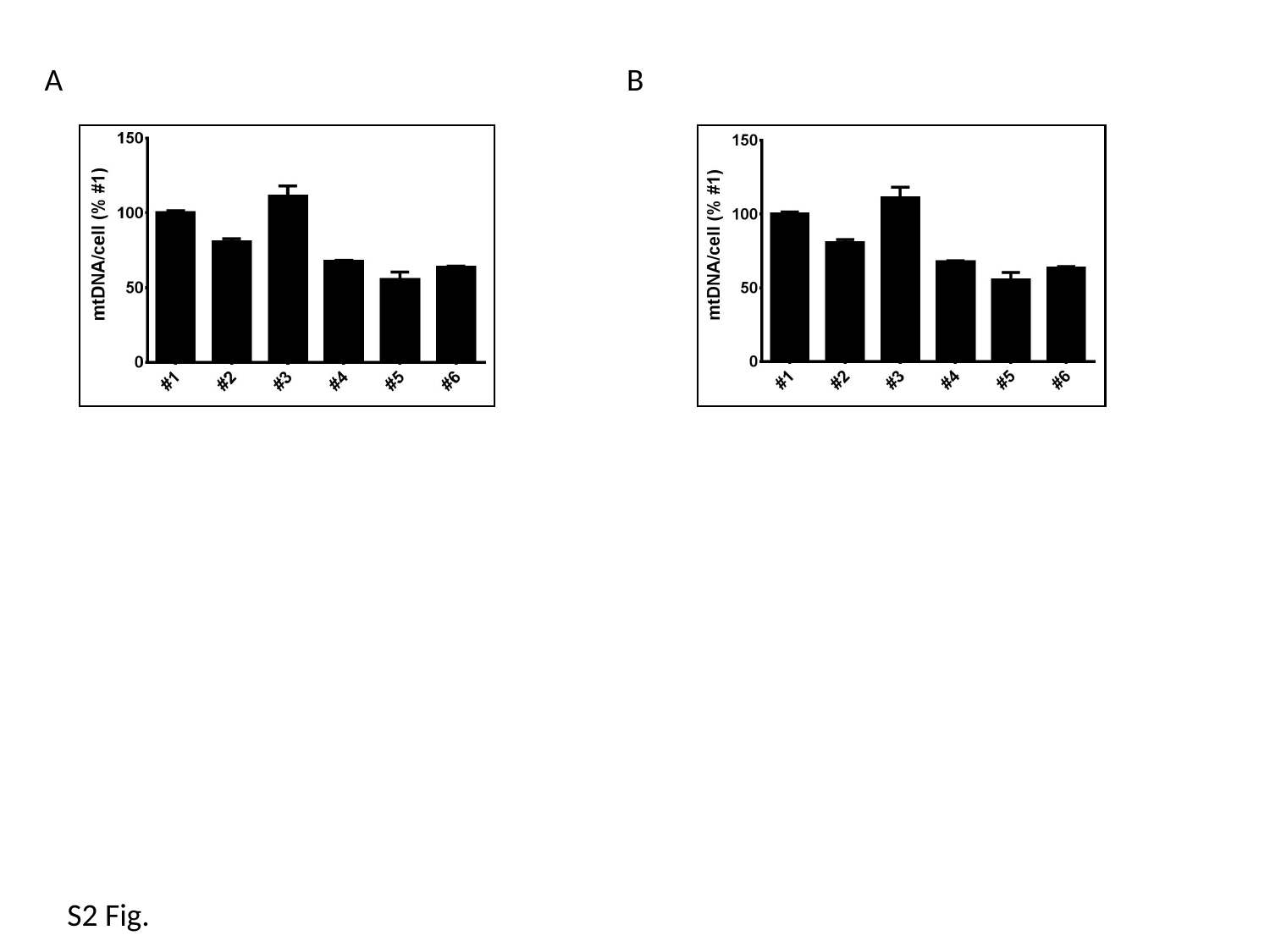

A
B
S2 Fig.

Supplement: S2 Fig — A, 4B6 cells were cloned, and mtDNA copy number was determined in six resulting subclones. B, subclones #1 was re-cloned, and mtDNA copy number was determined in 5 resulting subclones. (PPTX) [file pone.0152705.s002.pptx]

## Slide 1
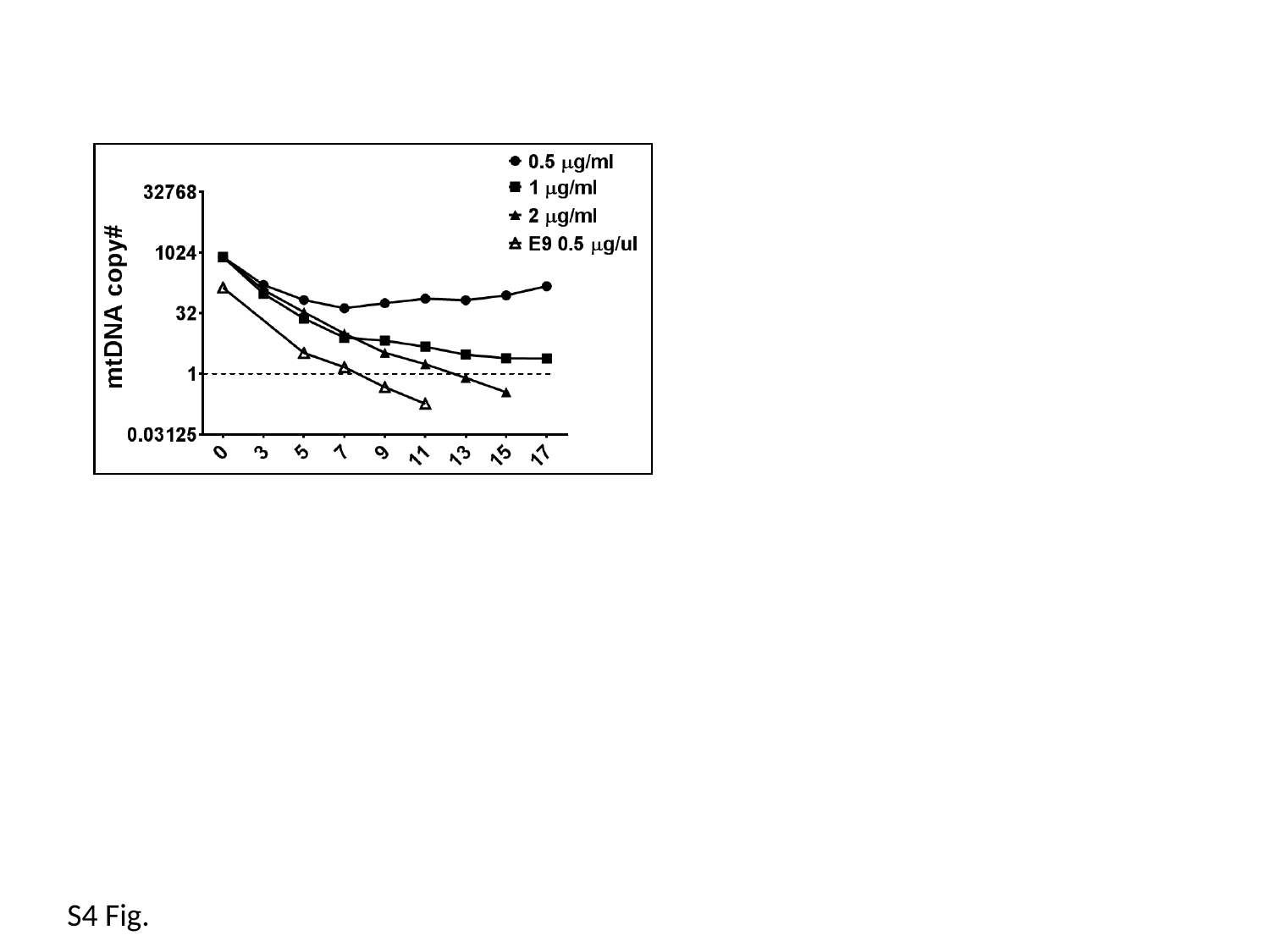

S4 Fig.

Supplement: S4 Fig — Parental 3T3#52 and its derivatives D4 and E9, in which mtDNA replication is supported by LigA were grown in the presence of indicated EtBr concentrations. A fraction of cells was removed at regular intervals, and mtDNA copy number was determined by qPCR. (PPTX) [file pone.0152705.s004.pptx]
