# Supplementary material for: Presequence-Independent Mitochondrial Import of DNA Ligase Facilitates Establishment of Cell Lines with Reduced mtDNA Copy Number
Source: PLoS One. 2016 Mar 31;11(3):e0152705. doi: 10.1371/journal.pone.0152705 (PMC4816344; doi:10.1371/journal.pone.0152705)
Supplement: S3 Fig — A, Deletions in the Lig3 exon 1 found in a clone with elevated mtDNA copy number. B and C, Deletions in the Lig3 exons 1 and 8, respectively, found in clones with reduced mtDNA copy number. Blue and underlined are gRNA targets, purple and underlined, sequences from an allele containing two in-frame deletions. Ter, premature translation termination, green and underlined AAG, a codon for active site lysine in exon 8. H, Reduced mtDNA copy number phenotype is stable over at least 3 weeks in clones with targeted exon 8. Clones #1, 2, 3 and 4 (Fig 6B) were grown in media supplemented with uridine and pyruvate, and mtDNA copy number was re-measured. (PPTX) [file pone.0152705.s003.pptx]

## Slide 1
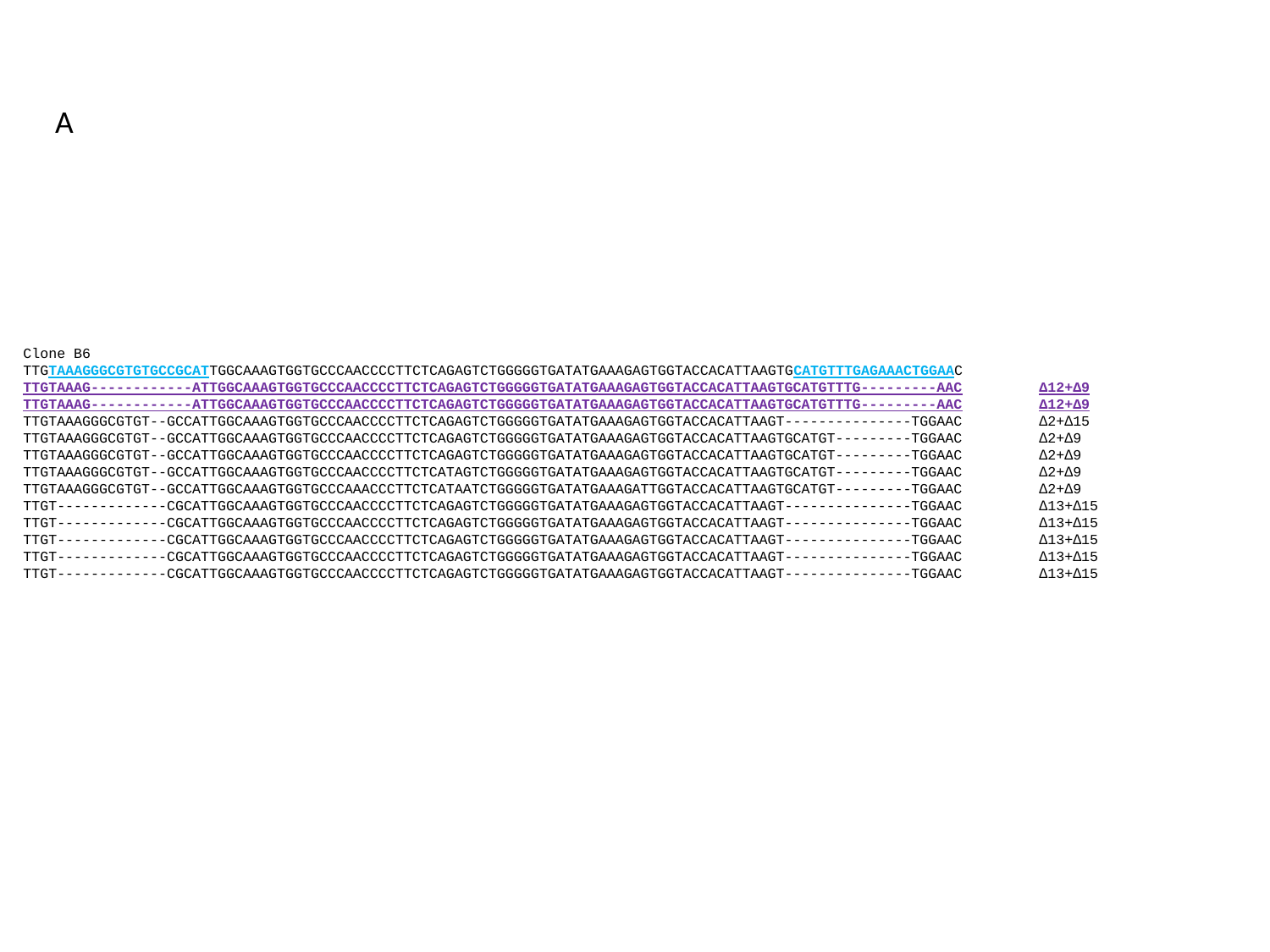

A
Clone B6
TTGTAAAGGGCGTGTGCCGCATTGGCAAAGTGGTGCCCAACCCCTTCTCAGAGTCTGGGGGTGATATGAAAGAGTGGTACCACATTAAGTGCATGTTTGAGAAACTGGAAC
TTGTAAAG------------ATTGGCAAAGTGGTGCCCAACCCCTTCTCAGAGTCTGGGGGTGATATGAAAGAGTGGTACCACATTAAGTGCATGTTTG---------AAC	∆12+∆9
TTGTAAAG------------ATTGGCAAAGTGGTGCCCAACCCCTTCTCAGAGTCTGGGGGTGATATGAAAGAGTGGTACCACATTAAGTGCATGTTTG---------AAC	∆12+∆9
TTGTAAAGGGCGTGT--GCCATTGGCAAAGTGGTGCCCAACCCCTTCTCAGAGTCTGGGGGTGATATGAAAGAGTGGTACCACATTAAGT---------------TGGAAC	∆2+∆15
TTGTAAAGGGCGTGT--GCCATTGGCAAAGTGGTGCCCAACCCCTTCTCAGAGTCTGGGGGTGATATGAAAGAGTGGTACCACATTAAGTGCATGT---------TGGAAC	∆2+∆9
TTGTAAAGGGCGTGT--GCCATTGGCAAAGTGGTGCCCAACCCCTTCTCAGAGTCTGGGGGTGATATGAAAGAGTGGTACCACATTAAGTGCATGT---------TGGAAC	∆2+∆9
TTGTAAAGGGCGTGT--GCCATTGGCAAAGTGGTGCCCAACCCCTTCTCATAGTCTGGGGGTGATATGAAAGAGTGGTACCACATTAAGTGCATGT---------TGGAAC	∆2+∆9
TTGTAAAGGGCGTGT--GCCATTGGCAAAGTGGTGCCCAAACCCTTCTCATAATCTGGGGGTGATATGAAAGATTGGTACCACATTAAGTGCATGT---------TGGAAC	∆2+∆9
TTGT-------------CGCATTGGCAAAGTGGTGCCCAACCCCTTCTCAGAGTCTGGGGGTGATATGAAAGAGTGGTACCACATTAAGT---------------TGGAAC	∆13+∆15
TTGT-------------CGCATTGGCAAAGTGGTGCCCAACCCCTTCTCAGAGTCTGGGGGTGATATGAAAGAGTGGTACCACATTAAGT---------------TGGAAC	∆13+∆15
TTGT-------------CGCATTGGCAAAGTGGTGCCCAACCCCTTCTCAGAGTCTGGGGGTGATATGAAAGAGTGGTACCACATTAAGT---------------TGGAAC	∆13+∆15
TTGT-------------CGCATTGGCAAAGTGGTGCCCAACCCCTTCTCAGAGTCTGGGGGTGATATGAAAGAGTGGTACCACATTAAGT---------------TGGAAC	∆13+∆15
TTGT-------------CGCATTGGCAAAGTGGTGCCCAACCCCTTCTCAGAGTCTGGGGGTGATATGAAAGAGTGGTACCACATTAAGT---------------TGGAAC	∆13+∆15

## Slide 2
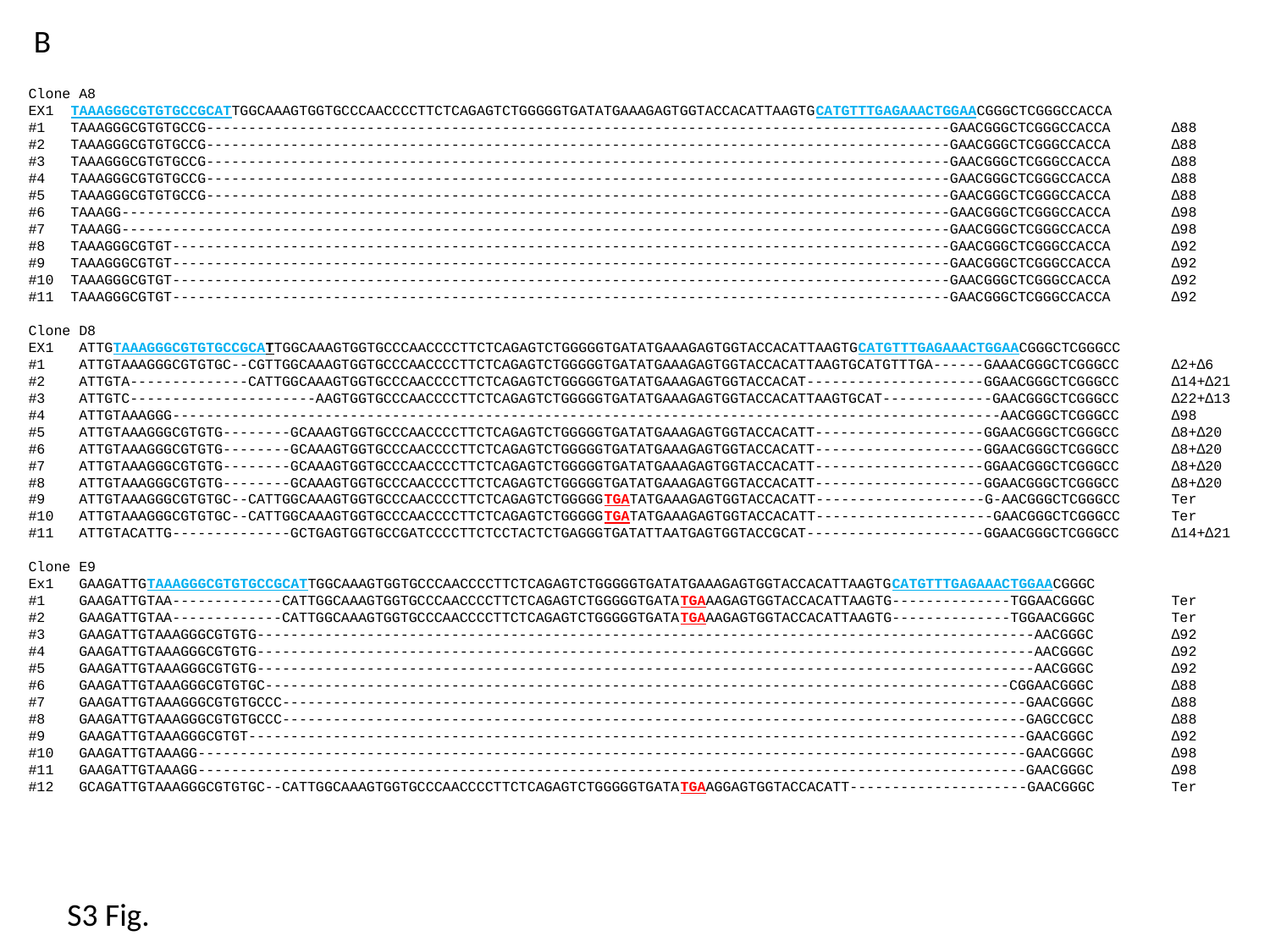

B
Clone A8
EX1 TAAAGGGCGTGTGCCGCATTGGCAAAGTGGTGCCCAACCCCTTCTCAGAGTCTGGGGGTGATATGAAAGAGTGGTACCACATTAAGTGCATGTTTGAGAAACTGGAACGGGCTCGGGCCACCA
#1 TAAAGGGCGTGTGCCG----------------------------------------------------------------------------------------GAACGGGCTCGGGCCACCA	∆88
#2 TAAAGGGCGTGTGCCG----------------------------------------------------------------------------------------GAACGGGCTCGGGCCACCA	∆88
#3 TAAAGGGCGTGTGCCG----------------------------------------------------------------------------------------GAACGGGCTCGGGCCACCA	∆88
#4 TAAAGGGCGTGTGCCG----------------------------------------------------------------------------------------GAACGGGCTCGGGCCACCA	∆88
#5 TAAAGGGCGTGTGCCG----------------------------------------------------------------------------------------GAACGGGCTCGGGCCACCA	∆88
#6 TAAAGG--------------------------------------------------------------------------------------------------GAACGGGCTCGGGCCACCA	∆98
#7 TAAAGG--------------------------------------------------------------------------------------------------GAACGGGCTCGGGCCACCA	∆98
#8 TAAAGGGCGTGT--------------------------------------------------------------------------------------------GAACGGGCTCGGGCCACCA	∆92
#9 TAAAGGGCGTGT--------------------------------------------------------------------------------------------GAACGGGCTCGGGCCACCA	∆92
#10 TAAAGGGCGTGT--------------------------------------------------------------------------------------------GAACGGGCTCGGGCCACCA	∆92
#11 TAAAGGGCGTGT--------------------------------------------------------------------------------------------GAACGGGCTCGGGCCACCA	∆92
Clone D8
EX1 ATTGTAAAGGGCGTGTGCCGCATTGGCAAAGTGGTGCCCAACCCCTTCTCAGAGTCTGGGGGTGATATGAAAGAGTGGTACCACATTAAGTGCATGTTTGAGAAACTGGAACGGGCTCGGGCC
#1 ATTGTAAAGGGCGTGTGC--CGTTGGCAAAGTGGTGCCCAACCCCTTCTCAGAGTCTGGGGGTGATATGAAAGAGTGGTACCACATTAAGTGCATGTTTGA------GAAACGGGCTCGGGCC	∆2+∆6
#2 ATTGTA--------------CATTGGCAAAGTGGTGCCCAACCCCTTCTCAGAGTCTGGGGGTGATATGAAAGAGTGGTACCACAT---------------------GGAACGGGCTCGGGCC	∆14+∆21
#3 ATTGTC----------------------AAGTGGTGCCCAACCCCTTCTCAGAGTCTGGGGGTGATATGAAAGAGTGGTACCACATTAAGTGCAT-------------GAACGGGCTCGGGCC	∆22+∆13
#4 ATTGTAAAGGG--------------------------------------------------------------------------------------------------AACGGGCTCGGGCC	∆98
#5 ATTGTAAAGGGCGTGTG--------GCAAAGTGGTGCCCAACCCCTTCTCAGAGTCTGGGGGTGATATGAAAGAGTGGTACCACATT--------------------GGAACGGGCTCGGGCC	∆8+∆20
#6 ATTGTAAAGGGCGTGTG--------GCAAAGTGGTGCCCAACCCCTTCTCAGAGTCTGGGGGTGATATGAAAGAGTGGTACCACATT--------------------GGAACGGGCTCGGGCC	∆8+∆20
#7 ATTGTAAAGGGCGTGTG--------GCAAAGTGGTGCCCAACCCCTTCTCAGAGTCTGGGGGTGATATGAAAGAGTGGTACCACATT--------------------GGAACGGGCTCGGGCC	∆8+∆20
#8 ATTGTAAAGGGCGTGTG--------GCAAAGTGGTGCCCAACCCCTTCTCAGAGTCTGGGGGTGATATGAAAGAGTGGTACCACATT--------------------GGAACGGGCTCGGGCC	∆8+∆20
#9 ATTGTAAAGGGCGTGTGC--CATTGGCAAAGTGGTGCCCAACCCCTTCTCAGAGTCTGGGGGTGATATGAAAGAGTGGTACCACATT--------------------G-AACGGGCTCGGGCC	Ter
#10 ATTGTAAAGGGCGTGTGC--CATTGGCAAAGTGGTGCCCAACCCCTTCTCAGAGTCTGGGGGTGATATGAAAGAGTGGTACCACATT---------------------GAACGGGCTCGGGCC	Ter
#11 ATTGTACATTG--------------GCTGAGTGGTGCCGATCCCCTTCTCCTACTCTGAGGGTGATATTAATGAGTGGTACCGCAT---------------------GGAACGGGCTCGGGCC	∆14+∆21
Clone E9
Ex1 GAAGATTGTAAAGGGCGTGTGCCGCATTGGCAAAGTGGTGCCCAACCCCTTCTCAGAGTCTGGGGGTGATATGAAAGAGTGGTACCACATTAAGTGCATGTTTGAGAAACTGGAACGGGC
#1 GAAGATTGTAA-------------CATTGGCAAAGTGGTGCCCAACCCCTTCTCAGAGTCTGGGGGTGATATGAAAGAGTGGTACCACATTAAGTG--------------TGGAACGGGC	Ter
#2 GAAGATTGTAA-------------CATTGGCAAAGTGGTGCCCAACCCCTTCTCAGAGTCTGGGGGTGATATGAAAGAGTGGTACCACATTAAGTG--------------TGGAACGGGC	Ter
#3 GAAGATTGTAAAGGGCGTGTG--------------------------------------------------------------------------------------------AACGGGC	∆92
#4 GAAGATTGTAAAGGGCGTGTG--------------------------------------------------------------------------------------------AACGGGC	∆92
#5 GAAGATTGTAAAGGGCGTGTG--------------------------------------------------------------------------------------------AACGGGC	∆92
#6 GAAGATTGTAAAGGGCGTGTGC----------------------------------------------------------------------------------------CGGAACGGGC	∆88
#7 GAAGATTGTAAAGGGCGTGTGCCC----------------------------------------------------------------------------------------GAACGGGC	∆88
#8 GAAGATTGTAAAGGGCGTGTGCCC----------------------------------------------------------------------------------------GAGCCGCC	∆88
#9 GAAGATTGTAAAGGGCGTGT--------------------------------------------------------------------------------------------GAACGGGC	∆92
#10 GAAGATTGTAAAGG--------------------------------------------------------------------------------------------------GAACGGGC	∆98
#11 GAAGATTGTAAAGG--------------------------------------------------------------------------------------------------GAACGGGC	∆98
#12 GCAGATTGTAAAGGGCGTGTGC--CATTGGCAAAGTGGTGCCCAACCCCTTCTCAGAGTCTGGGGGTGATATGAAGGAGTGGTACCACATT---------------------GAACGGGC	Ter
S3 Fig.

## Slide 3
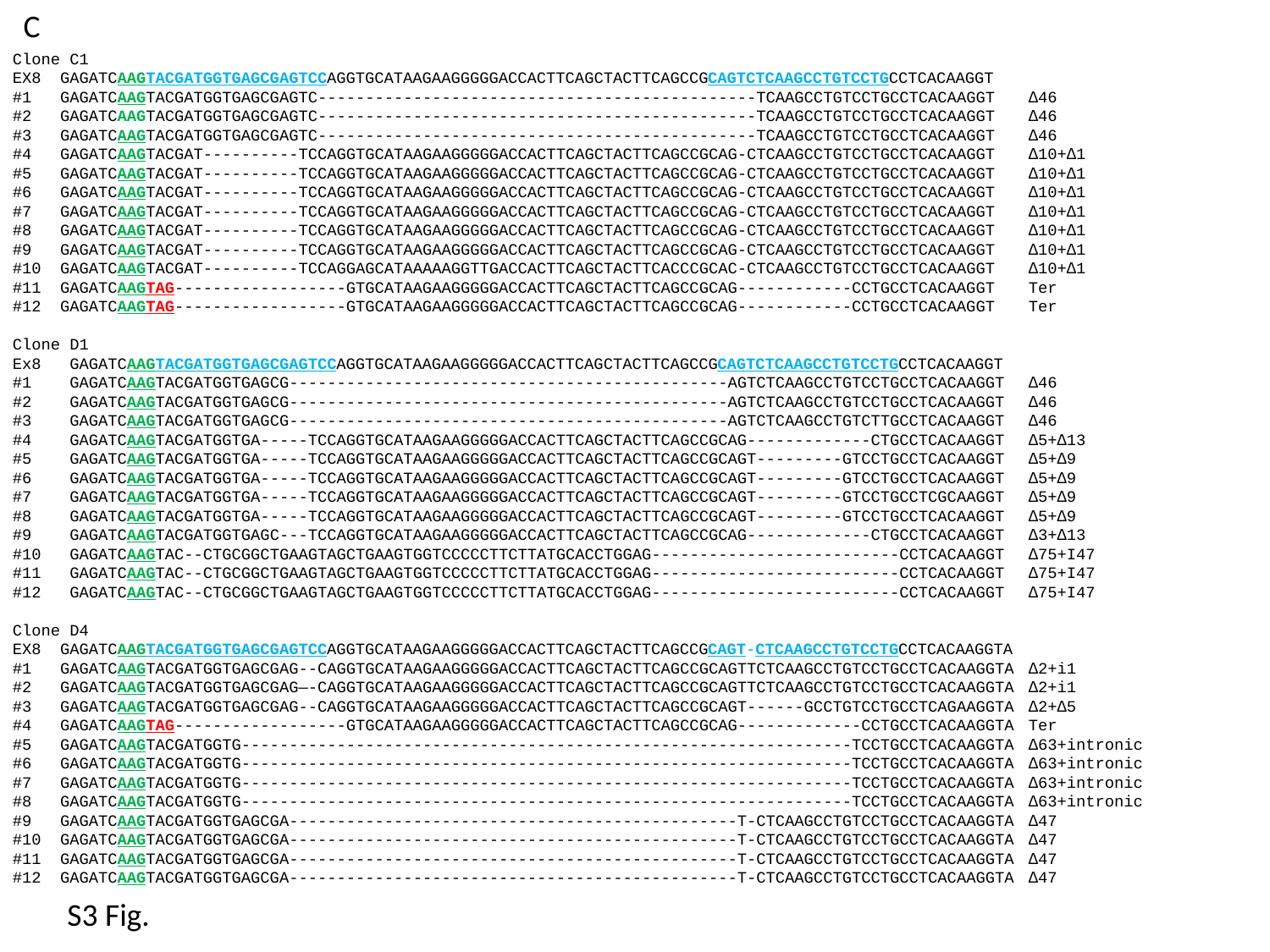

C
Clone C1
EX8 GAGATCAAGTACGATGGTGAGCGAGTCCAGGTGCATAAGAAGGGGGACCACTTCAGCTACTTCAGCCGCAGTCTCAAGCCTGTCCTGCCTCACAAGGT
#1 GAGATCAAGTACGATGGTGAGCGAGTC----------------------------------------------TCAAGCCTGTCCTGCCTCACAAGGT	∆46
#2 GAGATCAAGTACGATGGTGAGCGAGTC----------------------------------------------TCAAGCCTGTCCTGCCTCACAAGGT	∆46
#3 GAGATCAAGTACGATGGTGAGCGAGTC----------------------------------------------TCAAGCCTGTCCTGCCTCACAAGGT	∆46
#4 GAGATCAAGTACGAT----------TCCAGGTGCATAAGAAGGGGGACCACTTCAGCTACTTCAGCCGCAG-CTCAAGCCTGTCCTGCCTCACAAGGT	∆10+∆1
#5 GAGATCAAGTACGAT----------TCCAGGTGCATAAGAAGGGGGACCACTTCAGCTACTTCAGCCGCAG-CTCAAGCCTGTCCTGCCTCACAAGGT	∆10+∆1
#6 GAGATCAAGTACGAT----------TCCAGGTGCATAAGAAGGGGGACCACTTCAGCTACTTCAGCCGCAG-CTCAAGCCTGTCCTGCCTCACAAGGT	∆10+∆1
#7 GAGATCAAGTACGAT----------TCCAGGTGCATAAGAAGGGGGACCACTTCAGCTACTTCAGCCGCAG-CTCAAGCCTGTCCTGCCTCACAAGGT	∆10+∆1
#8 GAGATCAAGTACGAT----------TCCAGGTGCATAAGAAGGGGGACCACTTCAGCTACTTCAGCCGCAG-CTCAAGCCTGTCCTGCCTCACAAGGT	∆10+∆1
#9 GAGATCAAGTACGAT----------TCCAGGTGCATAAGAAGGGGGACCACTTCAGCTACTTCAGCCGCAG-CTCAAGCCTGTCCTGCCTCACAAGGT	∆10+∆1
#10 GAGATCAAGTACGAT----------TCCAGGAGCATAAAAAGGTTGACCACTTCAGCTACTTCACCCGCAC-CTCAAGCCTGTCCTGCCTCACAAGGT	∆10+∆1
#11 GAGATCAAGTAG------------------GTGCATAAGAAGGGGGACCACTTCAGCTACTTCAGCCGCAG------------CCTGCCTCACAAGGT	Ter
#12 GAGATCAAGTAG------------------GTGCATAAGAAGGGGGACCACTTCAGCTACTTCAGCCGCAG------------CCTGCCTCACAAGGT	Ter
Clone D1
Ex8 GAGATCAAGTACGATGGTGAGCGAGTCCAGGTGCATAAGAAGGGGGACCACTTCAGCTACTTCAGCCGCAGTCTCAAGCCTGTCCTGCCTCACAAGGT
#1 GAGATCAAGTACGATGGTGAGCG----------------------------------------------AGTCTCAAGCCTGTCCTGCCTCACAAGGT	∆46
#2 GAGATCAAGTACGATGGTGAGCG----------------------------------------------AGTCTCAAGCCTGTCCTGCCTCACAAGGT	∆46
#3 GAGATCAAGTACGATGGTGAGCG----------------------------------------------AGTCTCAAGCCTGTCTTGCCTCACAAGGT	∆46
#4 GAGATCAAGTACGATGGTGA-----TCCAGGTGCATAAGAAGGGGGACCACTTCAGCTACTTCAGCCGCAG-------------CTGCCTCACAAGGT	∆5+∆13
#5 GAGATCAAGTACGATGGTGA-----TCCAGGTGCATAAGAAGGGGGACCACTTCAGCTACTTCAGCCGCAGT---------GTCCTGCCTCACAAGGT	∆5+∆9
#6 GAGATCAAGTACGATGGTGA-----TCCAGGTGCATAAGAAGGGGGACCACTTCAGCTACTTCAGCCGCAGT---------GTCCTGCCTCACAAGGT	∆5+∆9
#7 GAGATCAAGTACGATGGTGA-----TCCAGGTGCATAAGAAGGGGGACCACTTCAGCTACTTCAGCCGCAGT---------GTCCTGCCTCGCAAGGT	∆5+∆9
#8 GAGATCAAGTACGATGGTGA-----TCCAGGTGCATAAGAAGGGGGACCACTTCAGCTACTTCAGCCGCAGT---------GTCCTGCCTCACAAGGT	∆5+∆9
#9 GAGATCAAGTACGATGGTGAGC---TCCAGGTGCATAAGAAGGGGGACCACTTCAGCTACTTCAGCCGCAG-------------CTGCCTCACAAGGT	∆3+∆13
#10 GAGATCAAGTAC--CTGCGGCTGAAGTAGCTGAAGTGGTCCCCCTTCTTATGCACCTGGAG--------------------------CCTCACAAGGT 	∆75+I47
#11 GAGATCAAGTAC--CTGCGGCTGAAGTAGCTGAAGTGGTCCCCCTTCTTATGCACCTGGAG--------------------------CCTCACAAGGT	∆75+I47
#12 GAGATCAAGTAC--CTGCGGCTGAAGTAGCTGAAGTGGTCCCCCTTCTTATGCACCTGGAG--------------------------CCTCACAAGGT	∆75+I47
Clone D4
EX8 GAGATCAAGTACGATGGTGAGCGAGTCCAGGTGCATAAGAAGGGGGACCACTTCAGCTACTTCAGCCGCAGT-CTCAAGCCTGTCCTGCCTCACAAGGTA
#1 GAGATCAAGTACGATGGTGAGCGAG--CAGGTGCATAAGAAGGGGGACCACTTCAGCTACTTCAGCCGCAGTTCTCAAGCCTGTCCTGCCTCACAAGGTA	∆2+i1
#2 GAGATCAAGTACGATGGTGAGCGAG—-CAGGTGCATAAGAAGGGGGACCACTTCAGCTACTTCAGCCGCAGTTCTCAAGCCTGTCCTGCCTCACAAGGTA	∆2+i1
#3 GAGATCAAGTACGATGGTGAGCGAG--CAGGTGCATAAGAAGGGGGACCACTTCAGCTACTTCAGCCGCAGT------GCCTGTCCTGCCTCAGAAGGTA	∆2+∆5
#4 GAGATCAAGTAG------------------GTGCATAAGAAGGGGGACCACTTCAGCTACTTCAGCCGCAG-------------CCTGCCTCACAAGGTA	Ter
#5 GAGATCAAGTACGATGGTG----------------------------------------------------------------TCCTGCCTCACAAGGTA	∆63+intronic
#6 GAGATCAAGTACGATGGTG----------------------------------------------------------------TCCTGCCTCACAAGGTA	∆63+intronic
#7 GAGATCAAGTACGATGGTG----------------------------------------------------------------TCCTGCCTCACAAGGTA	∆63+intronic
#8 GAGATCAAGTACGATGGTG----------------------------------------------------------------TCCTGCCTCACAAGGTA	∆63+intronic
#9 GAGATCAAGTACGATGGTGAGCGA-----------------------------------------------T-CTCAAGCCTGTCCTGCCTCACAAGGTA	∆47
#10 GAGATCAAGTACGATGGTGAGCGA-----------------------------------------------T-CTCAAGCCTGTCCTGCCTCACAAGGTA	∆47
#11 GAGATCAAGTACGATGGTGAGCGA-----------------------------------------------T-CTCAAGCCTGTCCTGCCTCACAAGGTA	∆47
#12 GAGATCAAGTACGATGGTGAGCGA-----------------------------------------------T-CTCAAGCCTGTCCTGCCTCACAAGGTA	∆47
S3 Fig.

## Slide 4
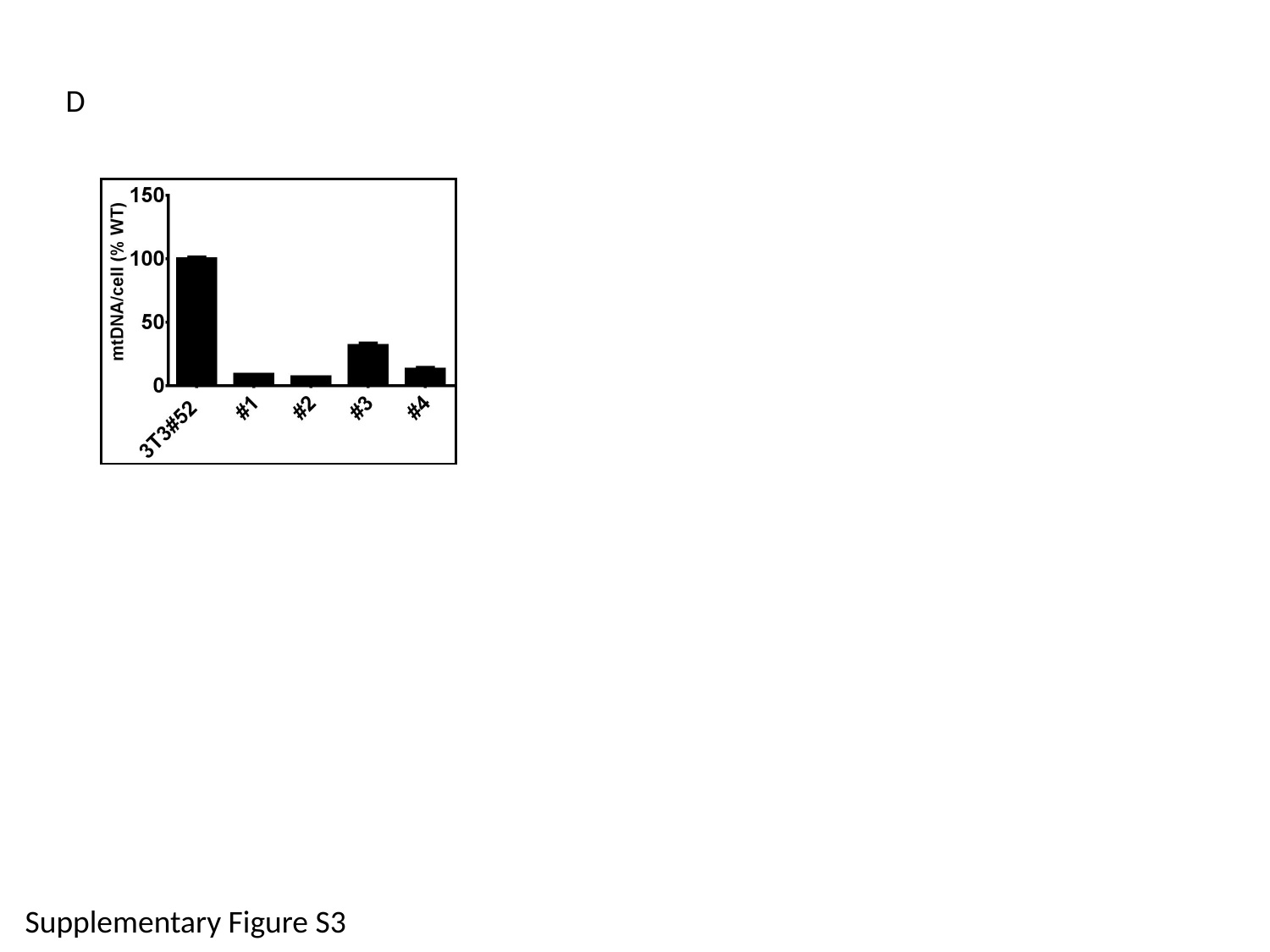

D
Supplementary Figure S3
