# Supplementary material for: Presequence-Independent Mitochondrial Import of DNA Ligase Facilitates Establishment of Cell Lines with Reduced mtDNA Copy Number
Source: PLoS One. 2016 Mar 31;11(3):e0152705. doi: 10.1371/journal.pone.0152705 (PMC4816344; doi:10.1371/journal.pone.0152705)
Supplement: S1 Table — (DOC) [file pone.0152705.s005.doc]

Supplementary Table S1. Oligonucleotides.

| Purpose | Name | Sequence | Fragment, bp |
| --- | --- | --- | --- |
| Diagnostics of the mLig 3 excision | Lig3-Del Lig3-5 | CAGTCGACGAGATGGCTCAGTGGTTAAGAGC GATGCGGCCGCAGCCAAGTGTGAATATACAGC | 560 |
| Diagnostics of the mLig3^flox/flox^ | Lig3-5 Lig3-8 | GATGCGGCCGCAGCCAAGTGTGAATATACAGC CAGTCGACAGGGAGCTTGGGACGGATGC | 555 |
| Lig3 exon1 sgRNA#3F Lig3 exon1 sgRNA#3R | Ex1#3F Ex1#3R | accgTAAAGGGCGTGTGCCGCAT aaacATGCGGCACACGCCCTTTA | N/A |
| Lig3 exon1 sgRNA#4F Lig3 exon1 sgRNA#4R | Ex1#4F Ex1#4R | accgCATGTTTGAGAAACTGGAA aaacTTCCAGTTTCTCAAACATG | N/A |
| Lig3 exon8 sgRNA#3F Lig3 exon8 sgRNA#3R | Ex1#3F Ex1#3R | accgTACGATGGTGAGCGAGTCC aaacGGACTCGCTCACCATCGTA | N/A |
| Multiplex diagnostics of CRISPR-Cas9 induced deletions in Lig3, exon 1 | Ex1R Ex1F1 Ex1F2 Ex1F3 | CCAAGAAGGATGCACAGAGAAA GTAAAGGGCGTGTGCCG GCTCTCCAGAGAGGTCATCTAA ACATTAAGTGCATGTTTGAGAAACTG | 311 476 230 |
| Multiplex diagnostics of CRISPR-Cas9 induced deletions in Lig3, exon 8 | Ex8R Ex8F1 Ex8F2 Ex8F3 | TTGTCTCAGGCAGCTCTTTC CAAGTACGATGGTGAGCGAG CATTTGCTTTCTCCATCCCAAG GCTACTTCAGCCGCAGT | 331 418 281 |
| Colony amplification for sequencing deletions in Lig 3 | dPvu2  dPvu3 | TGAGCGAGGAAGCGGAAGAG  TCAGGCTGCGCAACTGTTGG |  |
| Lig A qPCR | F R | TGCCAGTGAGTTGACCTTAATC AAGAAGTGCTGGCGTTCTATC | 95 |
| Lig A cloning | E.c.ligF E.c.ligR | Gcgaattcgccaccatggaatcaatcgaacaaca GCTCTAGAGTCAGCTACCCAGCAAACGCA | 2039 |
| Diagnostics of ρ^0^ phenotype in mouse cells | mMitF mMitR mNucF mNucR | AAAGCATCTGGCCTACACCCAGAA ACCCTCGTTTAGCCGTTCATGCTA CCACGTGCTCTGTATGAGATT ATGCTGGCTTATCTGTTCCTT | 1041  636 |
| Diagnostics of the FRT-MTS OTC-FRT excision | F R | CGCCTCAATCCTCCCTTTATC CTACACGTTGAGTAGGCGAATC | 497/338 |
