# Supplementary figures and images for: Correction: Presequence-Independent Mitochondrial Import of DNA Ligase Facilitates Establishment of Cell Lines with Reduced mtDNA Copy Number
Source: PLoS One. 2016 May 24;11(5):e0156168. doi: 10.1371/journal.pone.0156168 (PMC4878746; doi:10.1371/journal.pone.0156168)

## Slide 1
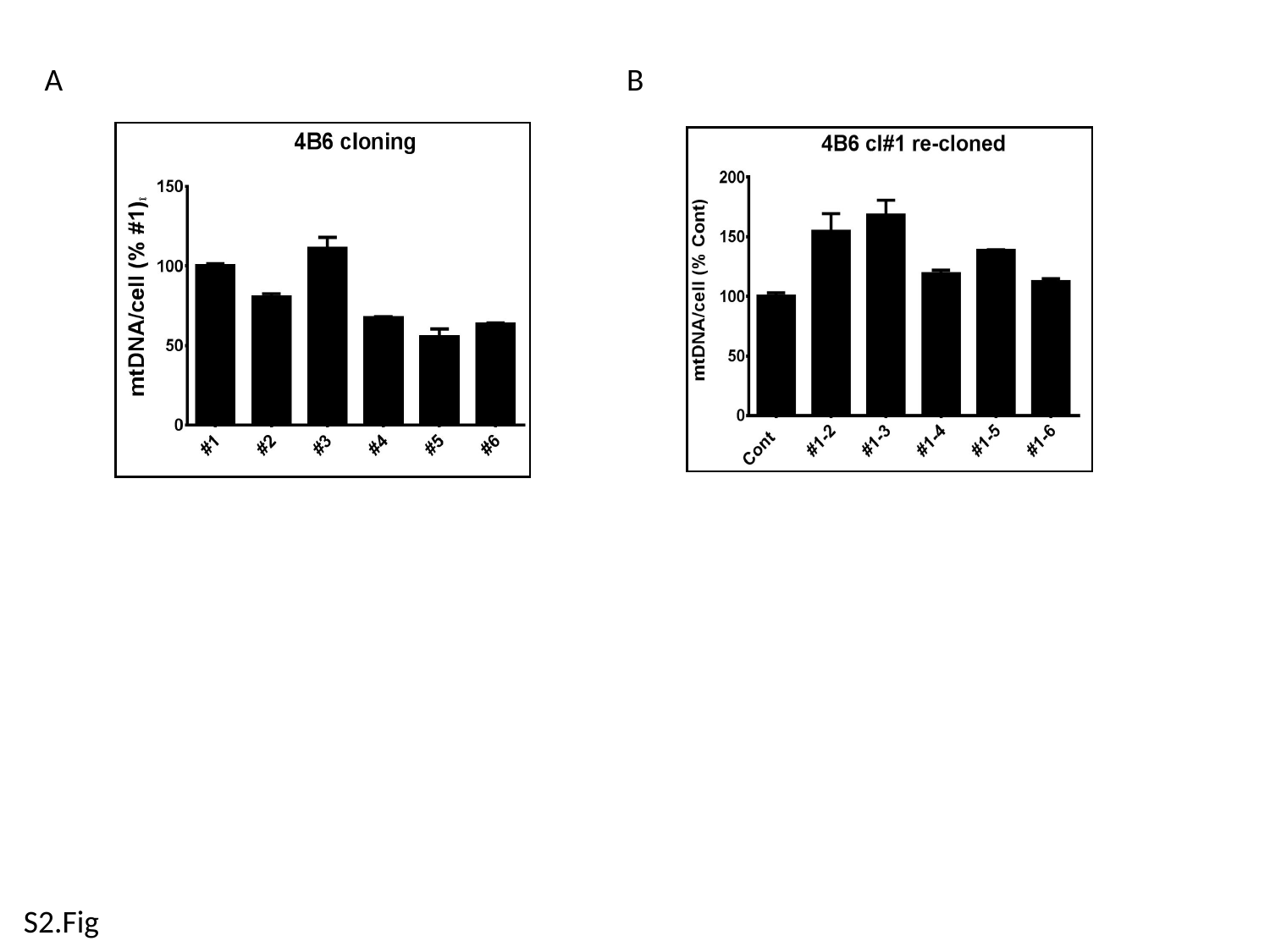

A
B
S2.Fig

Supplement: S2 Fig — A, 4B6 cells were cloned, and mtDNA copy number was determined in six resulting subclones. B, subclones #1 was re-cloned, and mtDNA copy number was determined in 5 resulting subclones. (PPTX) [file pone.0156168.s001.pptx]
